# Not all stress is equal: Examination stress and cortisol reactivity are associated with exploratory species-level variation but not broad restructuring of the gut microbiome

**DOI:** 10.1016/j.ynstr.2026.100855

**Published:** 2026-09-28

**Authors:** Connor B. O'Reilly, Timo Giesbrecht, John Tyson-Carr, Barry Murphy, Kendall Cagney, Luca Lenzi, Nicholas Fallon, Carl A. Roberts

**Affiliations:** a Department of Psychology, Institute of Population Health, University of Liverpool, . Eleanor Rathbone Building, Bedford Street South, L69 7ZA, UK; b Unilever Research and Development, Quarry Road East, Bebington, Port Sunlight, England, CH63 3JW, UK; c Centre for Genomic Research, LIV-SRF, University of Liverpool, Biosciences Building, Crown Street, Liverpool, L69 7ZB, UK

## Abstract

**Background:**

Psychological stress has been implicated in gut microbiome variation, but human evidence remains inconsistent and often does not distinguish exposure to stressful contexts from individual differences in physiological stress reactivity. This repeated-measures study examined whether naturalistic examination stress and post-awakening cortisol reactivity were associated with gut microbiome diversity, community composition, and species-level differential abundance.

**Methods:**

Thirty-nine healthy university students were assessed during examination and non-examination periods. At each assessment, participants completed validated measures of perceived stress, anxiety, affect, self-esteem, sleep, diet, and physical activity; provided salivary cortisol samples at awakening, 30 min, and 45 min post-awakening; and collected a stool sample for whole-metagenome shotgun sequencing. Post-awakening cortisol reactivity was indexed using area under the curve with respect to increase.

**Results:**

Examination stress was associated with increased perceived stress, state and trait anxiety, negative affect, poorer subjective sleep, and amplified post-awakening cortisol reactivity. Alpha and beta diversity of the gut microbiome did not differ between examination and non-examination periods, and within-subject changes in community composition were not associated with changes in cortisol reactivity. However, exploratory species-level analyses identified nominal selective microbial differences. Examination stress was associated with higher relative abundance of *Dialister massiliensis* and *Lawsonibacter asaccharolyticus*, alongside lower *Duodenibacillus massiliensis* and *Sutterella wadsworthensis*. Cortisol reactivity showed distinct associations with higher *Roseburia hominis* and *Ruminococcus bicirculans*, and lower *Methanobrevibacter smithii*. Within-subject increases in cortisol reactivity also covaried with higher *Dabirmavirus hominis*, *Phascolarctobacterium succinatutens* and lower *Coprococcus catus*.

**Conclusions:**

Short-term examination stress was associated with marked psychological and endocrine responses but was not accompanied by broad restructuring of the adult gut microbiome. Instead, exploratory findings suggest that examination stress and cortisol reactivity may be reflected in selective species-level microbial variation, supporting further longitudinal and functional investigation of stress-related microbiome dynamics.

## 1 Introduction

### 1.1 Background and rationale

The human gastrointestinal tract hosts a dense and metabolically active microbial ecosystem comprising trillions of microorganisms whose collective gene content far exceeds that of the human genome (Kurokawa et al., 2007; Sender et al., 2016). These communities contribute to host physiology through nutrient metabolism, short-chain fatty acid production, immune regulation, and maintenance of epithelial barrier integrity (Nicholson et al., 2012; Sekirov et al., 2010; Tremaroli and Bäckhed, 2012). In addition to these metabolic and immunological roles, gut microbes can influence neuroendocrine signalling through the production or modulation of bioactive metabolites and by interacting with host immune and neural pathways (Cryan et al., 2019; Cryan and Dinan, 2012).

Within this context, the microbiota–gut–brain axis (MGBA) describes a bidirectional network linking the gut microbiome with central nervous system (CNS) function via neural, endocrine, and immune mechanisms (Carabotti et al., 2015; Mayer, 2011). Signalling from the gut to the brain occurs through microbial metabolites, immune mediators, enteroendocrine pathways, and vagal afferents, while descending neural and endocrine outputs regulate gastrointestinal motility, permeability, immune tone, and nutrient availability, thereby shaping the microbial composition (Borre et al., 2014; Cryan et al., 2019; Dinan and Cryan, 2015). These pathways provide a plausible framework for bidirectional influence, whereby the gut microbiome may shape host behaviour and physiology, while psychological and physiological host states may, in turn, influence microbiome composition.

Psychological stress engages multiple physiological systems, with the hypothalamic–pituitary–adrenal (HPA) axis providing a central endocrine pathway through which stress-related signals influence immune and metabolic function. HPA-axis activation culminates in cortisol release, a glucocorticoid with widespread effects on immune activity, epithelial integrity, and metabolic regulation (Cain and Cidlowski, 2017; Sapolsky et al., 2000; Spencer and Deak, 2017).

Several mechanisms link HPA-axis activity to gut microbial ecology. Glucocorticoid receptors are expressed on intestinal epithelial and immune cells, allowing cortisol to influence barrier function, mucosal immune signalling, and inflammatory tone (Carabotti et al., 2015; Tetel et al., 2018). These host-mediated changes may alter the intestinal environment and contribute to shifts in microbial composition (Burokas et al., 2015; Cryan et al., 2019). Conversely, microbial metabolites and immune mediators can influence central stress-regulatory systems through immune and vagal afferent pathways, supporting bidirectional communication within the microbiota–gut–brain axis (Cryan et al., 2019; Rusch et al., 2023). Preclinical studies further show that gut microbiota can shape stress-related physiology (Bravo et al., 2011; Neufeld et al., 2011; Sudo et al., 2004). More specifically, germ-free mice show exaggerated HPA-axis responses to stress that can be modified by microbial colonisation (Sudo et al., 2004), while ingestion of *Lactobacillus rhamnosus* altered stress-related behaviour, central GABA-receptor expression, and corticosterone responses through a vagus-dependent mechanism in mice (Bravo et al., 2011).

However, translation to human populations has produced inconsistent results. A recent systematic review reported heterogeneous associations between psychological stress and gut microbiome diversity, with limited and inconsistent evidence for alpha- or beta-diversity differences (Ma et al., 2023). Although cortisol dynamics have been associated with gut microbial diversity or composition in some studies, particularly in early life (Keskitalo et al., 2021; Rosin et al., 2021), adult studies show mixed findings at the level of global community structure. For example, Karner et al. (2026) reported that higher alpha diversity was associated with greater perceived stress and salivary cortisol reactivity following acute stress, whereas Delgadillo et al. (2025) found higher alpha diversity among adults reporting lower perceived stress. These contrasting findings indicate that stress-related diversity effects are not directionally consistent across adult studies.

Despite this, there is emerging evidence to indicate that stress-related microbiome variation may extend beyond global diversity metrics to include taxon-level differences in specific microbial taxa. Across studies, higher psychological stress has been linked to reduced Lachnospira, Phascolarctobacterium, Sutterella, and Veillonella, and increased Methanobrevibacter and Roseburia, although effect directions and stress-type specificity vary across cohorts (Ma et al., 2023). Associations between cortisol measures and gut microbial taxa have also been reported in human studies, including evidence that cortisol response to acute stress is associated with differential abundance of specific bacterial taxa, although findings remain limited and heterogeneous (Almand et al., 2022; Hantsoo et al., 2018). Evidence from probiotic intervention studies further supports a taxon-focused approach, as targeted manipulation of bacterial strains has been associated with altered cortisol output and stress-related outcomes (Kato-Kataoka et al., 2016; Messaoudi et al., 2011; Venkataraman et al., 2021; Zhu et al., 2023).

Cortisol secretion follows a pronounced circadian rhythm, with elevated levels in the early morning and a gradual decline across the day (Dickmeis, 2009; Weitzman et al., 1971). Superimposed on this rhythm is the cortisol awakening response (CAR), a rapid rise in cortisol concentrations within the first 30–45 min after awakening (Schmidt-Reinwald et al., 1999). The CAR is a robust and reproducible phenomenon that is partly independent of basal diurnal cortisol secretion (Bouma et al., 2009; Clow et al., 2010; Stalder et al., 2016). Rather than indexing stress exposure directly, the CAR is widely interpreted as reflecting anticipatory physiological mobilisation for daily demands and is sensitive to individual differences in HPA-axis regulation and stress responsivity (Fries et al., 2009; Powell and Schlotz, 2012; Stalder et al., 2016). These characteristics make the CAR a useful marker for examining whether variation in HPA-axis function is associated with immune, metabolic, and microbial processes.

### 1.2 Aims and hypotheses

The present study employed a naturalistic quasi-experimental repeated-measures design comparing a period of examination-stress to a non-stress control period. We used whole-metagenome shotgun sequencing to determine whether sustained exposure to real-world psychological stress is associated with changes in gut microbiome structure, psychological wellbeing, and HPA axis reactivity. Microbiome structure was assessed using alpha diversity, beta diversity, and species-level abundance. Psychological wellbeing was indexed by subjective stress, anxiety, affect, and self-esteem. Physiological stress responsivity was indexed by CAR dynamics. We further examined whether individual differences in CAR reactivity were associated with selective microbial variation, whether CAR reactivity moderated stress-related abundance differences, and whether within-subject changes in CAR reactivity covaried with within-subject changes in species abundance. This framework allowed us to distinguish broad condition-related effects on global microbiome structure from exploratory species-level associations with physiological stress reactivity.

First, we hypothesised that examination stress would be associated with increased perceived stress, anxiety, and negative affect, alongside poorer sleep, diet, and physical activity relative to the non-stress period. Examination stress was also expected to increase HPA axis reactivity, indexed by amplification of the CAR.

Second, we tested whether gut microbiome structure differed between the stress and non-stress condition, indexed by measures of alpha and beta diversity. We also examined whether within-person changes in these diversity metrics were associated with corresponding changes in physiological stress reactivity, indexed by CAR area under the curve with respect to increase (AUCi).

Finally, exploratory species-level differential abundance analyses examined whether stress condition and CAR AUCi functioned as dissociable predictors of microbial variation, whether their interaction accounted for additional taxon-level differences, and whether within-subject changes in cortisol reactivity covaried with within-subject changes in species abundance. This framework allowed us to distinguish microbial variation associated with contextual stress exposure, individual differences in cortisol responsivity, reactivity-dependent modulation of stress-related abundance differences and coordinated within-person changes in cortisol reactivity and microbial abundance.

## 2 Methods

### 2.1 Participants

A priori power calculations were conducted in G*Power 3.1 (Faul et al., 2007), informed by examination-stress studies reporting moderate-to-large within-subject changes in perceived stress and CAR indices (Preuβ et al., 2010; Weekes et al., 2006). Assuming a conservative moderate paired effect, dz = 0.50, α = .05, and 90% power, 36 complete participant pairs were required for perceived stress and cortisol outcomes. For microbiome analyses, power is highly dependent on effect size, data structure, and diversity metric, with methodological work suggesting that community-level beta-diversity effects often require approximately 40 samples per group or more for adequate detection (Kers and Saccenti, 2022). Accordingly, 40 undergraduate students from the University of Liverpool were recruited via volunteer sampling and screened using a structured online eligibility questionnaire. The study was approved by the University of Liverpool Central University Research Ethics Committee D (ethics approval number: 13091).

### 2.2 Inclusion and exclusion criteria

Participants were eligible if they were aged ≥18 years, fluent in English, enrolled at the University of Liverpool, scheduled to complete at least one on-campus written or oral examination during the assessment period and willing and able to provide salivary cortisol and stool samples at both time points.

Participants were excluded for current or previous mental health diagnosis; gastrointestinal disorders, including inflammatory bowel disease, Crohn's disease, or irritable bowel syndrome; other chronic physical health condition; BMI ≤18 or ≥35; use of antibiotics, antifungals, probiotics, or corticosteroids within two weeks of sampling; major dietary change in the preceding month; history of substance or alcohol use disorder; pregnancy or lactation; or acute illness at sampling. These criteria were defined a priori to minimise confounding of hypothalamic–pituitary–adrenal axis function and gut microbiome composition.

### 2.3 Procedure

Following screening, eligible participants attended an in-person induction session, provided written informed consent, completed demographic questionnaires, and height and weight were measured to calculate BMI. The researcher provided detailed verbal and written instructions for saliva and stool sampling, including demonstration and rehearsal. Participants received instruction booklets and manufacturer video links.

Sampling dates for stress and non-stress conditions were agreed pragmatically in collaboration with the participant. On each sampling day, participants initiated saliva collection upon awakening, completed subjective questionnaires immediately thereafter, and collected a stool sample within 24 h. Samples were returned in person or via secure drop-off and stored at −80 °C prior to processing.

### 2.4 Sample characteristics

One participant withdrew prior to completing both assessment conditions, yielding a final analytic sample of 39 participants (23 females, 57.5%; mean age = 21.63 years, SD = 3.93). Most participants identified as White (82.5%) and were full-time students (75%). Prescription medication use (12.5%) and hormonal contraceptive use among female participants (47.8%) were recorded for descriptive purposes and sensitivity analyses.

### 2.5 Study design

A longitudinal, quasi-experimental repeated-measures design was used. Each participant completed two assessment conditions: a stress condition, scheduled proximal to end-of-year academic examinations, and a non-stress condition, scheduled during a period without examination demands. Examination date, proximity to the nearest examination, and examination format were recorded. Stress-condition sampling occurred before participants’ final examination and within one week of a scheduled examination, with a mean proximity of 1.67 ± 1.98 (mean ± SD) days from the nearest examination. The non-stress condition was scheduled approximately 4–6 weeks before or after the stress condition to allow recovery from academic stressors, with a mean inter-condition interval of 39.26 ± 8.25 days.

Condition order was counterbalanced: 20 participants completed the non-stress condition first and 19 completed the stress condition first. Order was determined pragmatically according to recruitment timing and academic scheduling constraints.

### 2.6 Subjective and behavioural measures

Subjective and behavioural measures were collected to (i) verify that the examination period constituted a psychologically stressful context and (ii) characterise potential behavioural factors that could confound physiological and microbiome outcomes. All questionnaires were administered electronically via QualtricsXM and completed on the morning of biological sampling.

### 2.7 Perceived stress

Perceived stress over the preceding month was assessed using the 10-item Perceived Stress Scale (PSS-10; Cohen et al., 1983). Items assess the extent to which life situations are appraised as unpredictable, uncontrollable, and overwhelming. Responses are recorded on a 5-point Likert scale (0 = never to 4 = very often), yielding total scores from 0 to 40, with higher scores indicating greater perceived stress.

### 2.8 Anxiety

State and trait anxiety were assessed using the State–Trait Anxiety Inventory (STAI; Spielberger, 1983), comprising two 20-item subscales measuring transient state anxiety (STAI-S) and stable trait anxiety (STAI-T). Items are rated on 4-point Likert scales, with higher scores indicating greater anxiety. The STAI has demonstrated robust psychometric properties across clinical and non-clinical samples.

### 2.9 Affective state

Affective state was assessed using the Positive and Negative Affect Schedule (PANAS; Watson et al., 1988). Participants rated the extent to which they experienced 10 positive and 10 negative affective states using a 5-point scale ranging from very slightly or not at all to extremely. Separate positive and negative affect scores were computed.

### 2.10 Sleep quality

Sleep quality over the previous month was assessed using the Pittsburgh Sleep Quality Index (PSQI; Buysse et al., 1989). The PSQI yields seven component scores—subjective sleep quality, sleep latency, sleep duration, habitual sleep efficiency, sleep disturbances, sleep medication use, and daytime dysfunction—which are summed to produce a global score ranging from 0 to 21, with higher scores indicating poorer sleep quality.

### 2.11 Dietary intake

Dietary intake was assessed using the University of Leeds Short-Form Food Frequency Questionnaire (SFFFQ; Cleghorn et al., 2016). Participants reported the frequency of key food-group consumption over a typical week in the previous month. Outputs included estimated intake in grams/day and a Dietary Quality Score reflecting adherence to dietary recommendations. The Dietary Quality Score reflects adherence to UK dietary recommendations across five components: fruit, vegetables, oily fish, dietary fat, and non-milk extrinsic sugars, with higher scores indicating better overall dietary quality.

### 2.12 Physical activity

Physical activity was assessed using an adapted online version of the Global Physical Activity Questionnaire (GPAQ; Armstrong and Bull, 2006), which captures work-related, recreational, and transport activity, as well as sedentary behaviour. Weekly physical activity durations were capped at 16 h/day, and sedentary time was capped at 960 min/day. Values below the minimum measurable resolution of the instrument were treated as missing. Derived outcomes included total physical activity, moderate-to-vigorous physical activity, sedentary time, and energy expenditure. A systematic review and meta-analysis of international physical-activity questionnaires reported moderate criterion validity for moderate-to-vigorous physical activity and supported MVPA as the most informative self-reported activity outcome (Sember et al., 2020).

### 2.13 Cortisol awakening response (CAR)

#### 2.13.1 Biochemical analysis and assay reliability

Salivary cortisol concentrations were quantified in duplicate using a high-sensitivity competitive enzyme-linked immunosorbent assay (ELISA; Salimetrics LLC, State College, PA, USA), with mean duplicate values used for analysis. Intra-assay reliability was high, with a mean coefficient of variation (CV) of 2.4% (median = 1.7%); >98% of samples had CVs below 10%.

### 2.14 Sampling procedure

Salivary cortisol was collected immediately upon awakening and at +30 and + 45 min post-awakening using Salimetrics SalivaBio oral swabs. Participants placed the swab under the tongue for 2 min before sealing it in a pre-labelled swab storage tube.

A forced-awakening protocol using a smartphone alarm was employed to improve temporal accuracy. Actual sampling times were recorded contemporaneously in a collection logbook and prioritised for analysis. Participants were instructed to refrain from eating, drinking (excluding water), smoking, engaging in physical activity, or returning to sleep during the sampling period.

Samples were refrigerated immediately after collection and stored at −80 °C upon return. Of the 39 participants with complete paired study data, full CAR data were available for 35 participants.

### 2.15 Derived cortisol indices

Raw cortisol concentrations were used to derive multiple indices following established recommendations (Pruessner et al., 2003). Cortisol at awakening reflects basal cortisol concentration. Area under the curve with respect to ground (AUCg) indexes total cortisol secretion across the sampling window, whereas area under the curve with respect to increase (AUCi) indexes dynamic change in cortisol relative to baseline and is interpreted as physiological stress reactivity.

### 2.16 Gastrointestinal microbiome sampling

Stool samples were self-collected by participants using the OMNIgene®•GUT OM-200 collection system (DNA Genotek, Ottawa, Canada) in accordance with the manufacturer's instructions. Participants were provided with detailed written and verbal guidance and instructed to collect a single stool sample within 24 h of each assessment time point. The OMNIgene®•GUT stabilisation buffer permits preservation of microbial DNA at ambient temperature during short-term storage and transport, thereby minimising degradation and compositional shifts.

Samples were returned to the laboratory either in person or via Royal Mail First Class post, typically within 48 h of collection. Upon receipt, samples were stored at −80 °C until downstream processing.

### 2.17 DNA extraction, library preparation, and sequencing

. After thawing and homogenisation, 200 μL of liquid stool or approximately 50–100 mg of solid stool was added to BashingBead™ buffer in a ZymoBIOMICS BashingBead Lysis Rack and mechanically disrupted using a TissueLyser II for 5 min at 25 Hz. Following centrifugation at 2000 × g for 10 min, DNA was extracted from the supernatant using Quick-DNA™ Fecal/Soil Microbe 96 MagBead Kit (Zymo Research, CA, USA) on a KingFisher™ Flex automated platform. DNA concentration was quantified using the Qubit™ dsDNA High Sensitivity assay, and integrity was assessed using the Agilent Fragment Analyzer™.

Shotgun metagenomic libraries were prepared on a Mosquito HV liquid handler (SPT Labtech, UK) using the NEBNext® Ultra™ II FS DNA Library Prep Kit for Illumina (New England Biolabs, MA, USA) miniaturised to 1/10th manufacturer standard volumes, using 26 ng DNA as the inputIndexed libraries were PCR amplified for 10 cycles, pooled equimolarly, size selected to remove primers and fragments <300 bp, quantified by qPCR, and sequenced on an Illumina NovaSeq X Plus platform using 2 × 150 bp paired-end reads with a 1% PhiX spike-in. All study libraries were sequenced together within a single sequencing run, so there was no between-run sequencing batch structure corresponding to the stress and non-stress conditions.

Host-derived reads were removed using HoCoRT v1.2.2 (Rumbavicius et al., 2023). Taxonomic classification was performed using Kraken2 v2.1.3 with a confidence threshold of 0.2 (Wood et al., 2019), and abundance estimates were refined using Bracken v2.9 (Lu et al., 2017). Bracken species-level count tables were used for downstream analyses.

## 3 Statistical analyses

All analyses were conducted in R 4.5.1. The complete computational environment, including package versions, was archived using sessionInfo(); key packages included MaAsLin3 1.5.3, vegan 2.7-5, compositions 2.0-9, afex 1.5-1, emmeans 2.0.4, and lme4 2.0-1. The Zenodo repository contains the reproducible R script, archived session information, analysis data, and complete outputs for all primary, supplementary, and sensitivity models.

### 3.1 Subjective outcomes

Differences between stress and non-stress conditions were assessed using paired-samples tests (paired t-tests or non-parametric equivalents, as appropriate). As a supplementary sensitivity analysis, potential sex differences in these responses were examined using Condition × Sex models for perceived stress, state and trait anxiety, positive and negative affect, and self-esteem; the corresponding interaction effects and within-sex contrasts are reported in Supplementary Table S1.

### 3.2 Sleep, diet and physical activity

Sleep, diet, and physical activity outcomes were compared between the stress and non-stress conditions using paired-samples tests (paired t-tests or Wilcoxon signed-rank tests where appropriate). Analyses included PSQI component and global scores, SFFFQ food-group scores and the Dietary Quality Score, and GPAQ-derived activity and sedentary outcomes. Sample size varied for physical-activity outcomes where missing or implausible responses were excluded according to the prespecified plausibility criteria. Full descriptive statistics and condition comparisons for sleep, diet, and physical activity are reported in the Supplementary Materials (Table S2–S4).

### 3.3 Cortisol awakening response (CAR)

CAR trajectories were analysed using a 3 × 2 repeated-measures ANOVA with Time (awakening, +30 min, +45 min) and Condition (stress, non-stress) as within-subject factors. Greenhouse–Geisser corrections were applied where appropriate, and follow-up pairwise comparisons were conducted using estimated marginal means with Holm adjustment. Derived CAR indices were compared between conditions using paired-samples t-tests when within-subject difference scores were approximately normally distributed and Wilcoxon signed-rank tests otherwise. As a supplementary sensitivity analysis, potential sex differences in cortisol responses were examined using Condition × Sex and Time × Condition × Sex interaction terms for the CAR trajectory, together with Condition × Sex analyses of derived cortisol indices; corresponding interaction effects and within-sex contrasts are reported in Supplementary Table S5.

### 3.4 Microbiome analyses

Species-level count tables generated by Bracken were matched to participant metadata using exact sample identifiers, and samples with zero assigned reads were excluded. Paired alpha-diversity, beta-diversity, and within-subject change analyses were restricted to participants with one stress and one non-stress sample; 39 complete participant pairs (78 samples) were available for these paired analyses. The primary mixed-effects MaAsLin3 models retained all matched microbiome samples remaining after preprocessing, with model-specific sample and participant counts recorded in the reproducibility outputs.

For species-level differential-abundance, beta-diversity, and delta analyses, taxa were filtered once before modelling and retained if present in at least 15% of matched microbiome samples and if mean relative abundance was at least 1 × 10^−4^ (0.01%). This externally defined feature set contained 174 species-level taxa and was held fixed across the corresponding analyses. Alpha diversity was treated separately and calculated from the unfiltered species-level relative-abundance table using the vegan package (Oksanen et al., 2026); Shannon diversity, Simpson diversity, and observed richness were compared between conditions using paired Wilcoxon signed-rank tests. Associations between within-subject changes in alpha diversity and cortisol reactivity were examined using change-score linear models (Δdiversity ∼ ΔAUCi).

Beta diversity was assessed using Aitchison distance. Within the externally filtered feature set, counts were converted to relative abundances and zeros were replaced with one-half of the minimum observed non-zero relative abundance (with a lower bound of 1 × 10^−10^) before sample-wise centred log-ratio (CLR) transformation. Euclidean distance in CLR space was then used as Aitchison distance. Differences in community composition between conditions were tested using PERMANOVA with permutations constrained by participant identifier, and homogeneity of multivariate dispersion was assessed using PERMDISP. Within-participant Aitchison distance between conditions was regressed on the signed change in AUCi and the absolute magnitude of that change.

Species-level differential abundance was assessed using MaAsLin3 (Nickols et al., 2026). Following external filtering, the Bracken species-level count matrix was supplied directly to MaAsLin3. CLR normalization was performed internally by MaAsLin3, with no manual pseudocount or prior CLR transformation applied, and no additional prevalence or abundance filtering was performed within the software (normalization = "CLR", transform = "NONE"). Repeated observations were accounted for using a participant-level random intercept, with small_random_effects = TRUE. For models including cortisol reactivity, AUCi was entered as a continuous predictor and standardised internally by MaAsLin3 (standardize = TRUE). The following primary mixed-effects models were fitted:

Model 1 CLR abundance ∼ stress condition + (1 | ID)

Model 2 CLR abundance ∼ stress condition + AUCi + (1 | ID)

Model 3 CLR abundance ∼ stress condition × AUCi + (1 | ID)

An additional within-subject change model was fitted as an exploratory analysis to examine whether change in species-level composition covaried with change in cortisol reactivity. For this analysis, 0.5 was added to the externally filtered count matrix before manual sample-wise CLR transformation. Participant-level abundance change was then calculated as Stress − Non-stress CLR abundance, and cortisol change was calculated as Stress − Non-stress for the stored standardised AUCi variable (ΔAUCi_z). The resulting ΔCLR matrix was supplied to MaAsLin3 with normalization = "NONE", transform = "NONE", and standardize = FALSE. Because CLR values express each taxon relative to the geometric mean of the retained community, ΔCLR represents a within-participant change in relative log-ratio position rather than an absolute change in taxon abundance.

Model 4 ΔCLR abundance ∼ ΔAUCi_z

Benjamini–Hochberg false-discovery-rate correction was applied across species-level tests. Associations with q < 0.05 were considered to survive multiple-testing correction. Associations with uncorrected p < .05 but q ≥ 0.05 are described as nominal/exploratory and are not interpreted as FDR-corrected statistical evidence.

### 3.5 Supplementary and sensitivity analyses

Additional analyses were conducted to distinguish exploratory species-level associations from robustness checks of the primary models. Supplementary univariate mixed-effects models examined associations between species-level abundance and cortisol AUCi and AUCg, perceived stress, state and trait anxiety, positive and negative affect, self-esteem, global sleep quality, moderate-to-vigorous physical activity, and sedentary behaviour. These analyses were treated as exploratory and hypothesis-generating and are reported in the Supplementary Materials (Table S6–S16).

Sensitivity analyses re-estimated the primary species-level models after adjustment for age, sex and BMI; separately for sleep quality, sedentary behaviour and vegetable intake; and with these lifestyle variables entered simultaneously. Additional sex-stratified models and a female hormonal-contraceptive-adjusted analysis were also conducted. Because these models were intended to assess robustness rather than provide a second inferential framework, interpretation focused primarily on whether the direction and approximate magnitude of the primary coefficients remained stable across alternative specifications, rather than on whether nominal *p* < .05 was retained after additional adjustment. A summary of coefficient stability for the primary stress-condition associations is provided in Table S17.

## 4 Results

### 4.1 Subjective stress, anxiety, and affect

Relative to the non-stress condition, the stress condition was associated with robust and internally consistent increases in perceived stress, State and Trait anxiety, and negative affect, alongside reductions in positive affect, indicating a clear psychological response to the quasi-experimental manipulation (Table 1). Self-esteem did not differ significantly between conditions, suggesting that the stress manipulation predominantly influenced transient psychological states rather than more stable self-evaluative constructs. Supplemental Condition × Sex analyses identified no significant interactions for perceived stress, state anxiety, trait anxiety, positive affect, negative affect, or self-esteem (Table S1), indicating that the subjective psychological response to examination stress did not differ significantly by sex. These results provide strong validation of the quasi-experimental manipulation and a contextual framework for interpreting downstream physiological and microbiome outcomes.

**Table 1:** Summary statistics and paired differences for subjective measures of stress and wellbeing across stress and non-stress conditions.

*Variable*	*Condition*	*N*	*Mean*	*SD*	*Paired difference (Stress − Non-stress)*	*SD*	*95% CI*	*p*
Perceived Stress	Non-stress	39	15.18	7.06	5.49	6.23	[3.47, 7.51]	< 0.001***
	Stress	39	20.67	7.60				
Trait Anxiety	Non-stress	39	40.26	11.11	6.79	8.74	[3.96, 9.63]	< 0.001***
	Stress	39	47.05	12.01				
State Anxiety	Non-stress	39	35.64	12.02	11.64	11.80	[7.82, 15.47]	< 0.001***
	Stress	39	47.28	13.96				
Positive Affect	Non-stress	39	29.97	7.87	−4.90	6.86	[-7.12, −2.67]	< 0.001***
	Stress	39	25.08	8.75				
Negative Affect	Non-stress	39	14.21	4.89	7.69	7.97	[5.11, 10.28]	< 0.001***
	Stress	39	21.90	9.05				
Self-Esteem	Non-stress	39	17.64	2.98	0.72	2.73	[-0.17, 1.60]	0.945
	Stress	39	18.36	3.22				

Notes. Values are presented as M ± SD. Paired differences are calculated as Stress − Non-stress condition scores. Statistical comparisons were conducted using paired-samples t-tests (one-tailed). Confidence intervals reflect 95% confidence levels. Higher scores indicate greater perceived stress, anxiety, and negative affect, and lower wellbeing where applicable.

Perceived Stress = Perceived Stress Scale (PSS-10); Trait Anxiety = State–Trait Anxiety Inventory–Trait (STAI-T); State Anxiety = State–Trait Anxiety Inventory–State (STAI-S); Positive Affect and Negative Affect = Positive and Negative Affect Schedule (PANAS); Self-Esteem = Rosenberg Self-Esteem Scale (RSE). p < .05*, p < .01**, p < .001***.

### 4.2 Sleep, diet, and physical activity

Sleep, diet and physical activity also differed selectively between assessment conditions (Table S2–S4). Global PSQI scores were higher during examination stress (M = 8.69, SD = 2.68) than during the non-stress condition (M = 7.03, SD = 2.06), corresponding to poorer subjective sleep quality (Stress − Non-stress = 1.67, p < .001). Vegetable-intake scores were lower during stress (M = 1.97, SD = 0.81) than non-stress (M = 2.21, SD = 0.77; difference = −0.23, p = .02), whereas the overall Dietary Quality Score did not differ significantly. Sedentary time was higher during stress (M = 394.36, SD = 158.44 min/day) than non-stress (M = 357.05, SD = 144.50 min/day; difference = 37.31 min/day, p = .04), while the remaining physical-activity indices did not show clear condition differences. Supplementary univariate analyses are reported in the Supplemental Materials (Table S6–S16).

### 4.3 Cortisol awakening response (CAR)

Salivary cortisol concentrations across the CAR under stress and non-stress conditions are shown in Fig. 1, with derived CAR metrics summarised in Table 2. Across both conditions, cortisol showed the expected post-awakening increase, with a significant main effect of Time, F(1.36, 46.28) = 43.81, p < .001, ηp^2^ = 0.56. Mean cortisol concentrations averaged across timepoints did not differ significantly between stress and non-stress conditions, F(1, 34) = 1.55, p = .221, ηp^2^ = 0.04. However, there was a significant Time × Condition interaction, F(1.66, 56.56) = 3.83, p = .035, ηp^2^ = 0.10, indicating that the CAR profile differed between conditions.

**Fig. 1:**
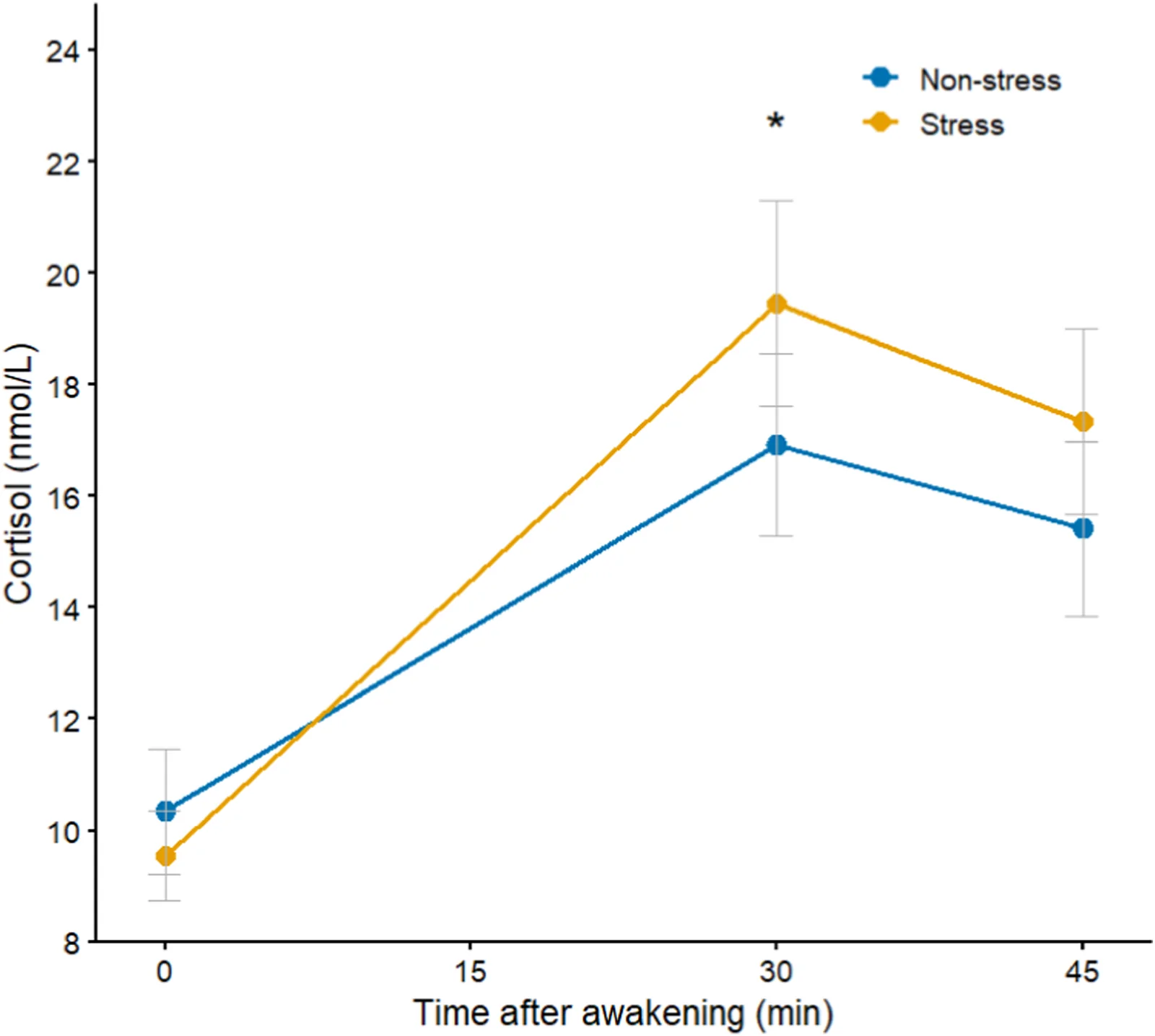
**CAR under non-stress and stress conditions.** Mean (±SE) salivary cortisol concentrations are shown at awakening (0 min), 30 min, and 45 min post-awakening for the non-stress and stress conditions. Cortisol increased following awakening in both conditions; however, the stress condition exhibited a significantly greater cortisol response at 30 min post-awakening. * indicates p < .05 for the between-condition comparison at 30 min. Analytic n = 35 participants with complete CAR data.

**Table 2:** Summary statistics and paired differences for CAR metrics across stress and non-stress conditions.

Variable	Non-stress M	Non-stress SD	Stress M	Stress SD	N	Paired Difference (Stress − Non-stress)	SD Difference	95% CI	p
Cortisol at awakening	10.34	6.65	9.54	4.71	35	−0.80	6.64	[-3.08, 1.48]	0.759
Cortisol at +30 min	16.93	9.67	19.46	10.95	35	2.53	7.26	[0.03, 5.02]	0.024*
Cortisol at +45 min	15.42	9.23	17.33	9.81	35	1.92	7.75	[-0.74, 4.58]	0.076
AUCg	653.89	359.84	729.35	371.57	35	75.46	250.73	[-10.67, 161.59]	0.042*
AUCi	186.47	198.57	286.73	247.00	35	100.26	245.64	[15.88, 184.64]	0.011*
Peak cortisol	17.52	9.54	20.32	11.11	35	2.81	7.56	[0.21, 5.41]	0.017*
Max increase	7.17	6.78	10.78	9.09	35	3.61	9.13	[0.47, 6.74]	0.013*

Note. Values are presented as means and standard deviations. Paired differences were calculated as stress minus non-stress condition values. For each metric, normality of within-subject difference scores was assessed using the Shapiro-Wilk test. Paired-samples t-tests were applied where difference scores were approximately normally distributed; otherwise, Wilcoxon signed-rank tests were used. Confidence intervals are one-sided 95% confidence intervals for the paired mean difference. AUCg = area under the curve with respect to ground; AUCi = area under the curve with respect to increase. p < .05*, p < .01**, p < .001***.

Holm-adjusted follow-up comparisons showed that cortisol concentrations did not differ significantly between conditions at awakening, t(34) = 0.71, p = .482, or at +45 min, t(34) = −1.46, p = .152. However, cortisol was significantly higher during the stress condition at +30 min, t(34) = −2.06, p = .047. Consistent with this pattern, directional paired comparisons of derived CAR metrics showed significantly greater AUCg, AUCi, peak cortisol, and maximum post-awakening increase during the stress condition. Overall, examination stress was associated with an amplified post-awakening cortisol response, particularly at the +30 min peak, rather than a general elevation in cortisol across all sampling points.

Sex-related sensitivity analyses provided no statistically significant evidence that the cortisol response to examination stress differed by sex. Neither the Condition × Sex interaction, F(1, 33) = 0.22, p = .641, ηp^2^ = 0.007, nor the Time × Condition × Sex interaction across the CAR, F(1.71, 56.36) = 2.19, p = .129, ηp^2^ = 0.062, was significant. Similarly, there was no Condition × Sex interaction for AUCg, F(1, 33) = 0.07, p = .796, ηp^2^ = 0.002. The corresponding interaction for AUCi was larger but remained non-significant, F(1, 33) = 2.92, p = .097, ηp^2^ = 0.081, providing no clear evidence of sex-specific differences in cortisol reactivity (Table S5).

### 4.4 Alpha diversity

As summarised in Table 3, alpha diversity metrics were comparable between stress and non-stress conditions. No significant within-subject differences were observed for Shannon diversity, Simpson diversity, or observed species richness.

**Table 3:** Alpha diversity metrics across stress and non-stress conditions.

Metric	Non-Stress		Stress		N	Wilcoxon V	*p*
	Mean	SD	Mean	SD			
Shannon diversity (H′)	2.74	0.71	2.81	0.71	39	457	0.35
Simpson diversity	0.81	0.17	0.82	0.17	39	411	0.77
Observed species richness	366.3	50.5	368.2	41.3	39	396	0.94

**Notes.** Values are presented as mean (*SD*). *N* reflects the number of participants contributing paired non-stress and stress samples. Differences between conditions were assessed using paired Wilcoxon signed-rank tests.

Within-subject changes in alpha diversity were not significantly associated with corresponding changes in cortisol reactivity (ΔAUCi). Change-score linear models indicated no evidence of an association between ΔAUCi and change in Shannon diversity (β = −0.044, SE = 0.057, p = .447), Simpson diversity (β = −0.015, SE = 0.012, p = .211), or observed species richness (β = −5.49, SE = 8.41, p = .518).

### 4.5 Beta diversity

Global gut microbiome community composition did not significantly differ between the non-stress and stress conditions. PERMANOVA based on Aitchison distances revealed no association between condition and beta diversity, F(1, 76) = 0.14, R^2^ = 0.0018, p = .443. There was likewise no evidence of differential multivariate dispersion by condition, F(1, 76) = 0.02, p = .883, suggesting that the observed null effect was not confounded by heterogeneity of variance. Ordination analyses (Fig. 2) supported this interpretation, with considerable overlap between stress and non-stress samples and no consistent directional shift in within-subject community composition.

**Fig. 2:**
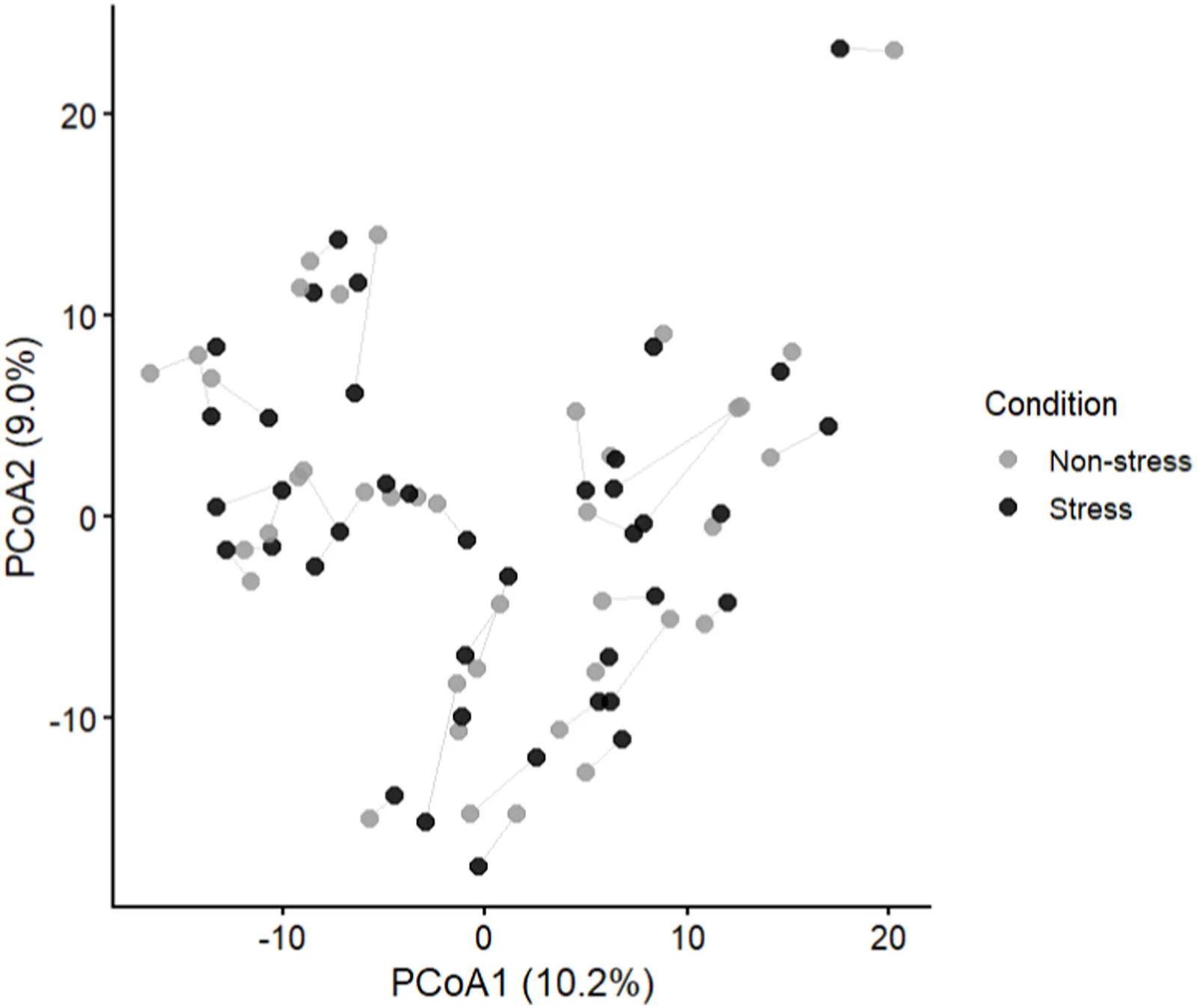
Paired principal coordinates analysis (PCoA) trajectories illustrating within-participant changes in gut microbiome co*mposition.* PCoA ordination is based on Aitchison distances calculated from centred log-ratio–transformed species-level relative abundance data. Each line connects paired non-stress and stress samples from the same participant, illustrating heterogeneous within-subject shifts in community composition without a consistent directional pattern across participants. Analytic n = 39 participants (78 paired microbiome samples).

Physiological stress reactivity was not associated with within-subject changes in global community composition. In the paired complete-case subset, paired Aitchison distance between stress and non-stress samples was unrelated to either the signed change in cortisol reactivity (ΔAUCi; b = −0.62, SE = 0.88, p = .487) or the absolute magnitude of cortisol change (|ΔAUCi|; b = −0.90, SE = 1.07, p = .407), indicating no evidence that cortisol reactivity explained within-subject beta-diversity shift.

### 4.6 Differential abundance associated with stress condition

In the primary model examining stress condition alone (Model 1), several bacterial taxa showed nominal differences in relative abundance between stress and non-stress conditions (Table 4). Stress exposure was associated with higher relative abundance of *Dialister massiliensis*, *Lawsonibacter asaccharolyticus*, *Dialister hominis*, *Oscillibacter hominis*, and *Oscillibacter* sp*. PEA192.* By contrast, lower relative abundance under stress was observed for *Duodenibacillus massiliensis*, *Sutterella wadsworthensis*, *Phocaeicola coprophilus*, *Blautia obeum*, and *Phascolarctobacterium* sp*. Marseille-Q4147*. Several taxa identified in the primary stress condition model were also observed across the supplementary univariate models (Table S6–S16). In particular, *Duodenibacillus massiliensis*, *Lawsonibacter asaccharolyticus*, *Phascolarctobacterium* sp*. Marseille-Q4147*, *Sutterella wadsworthensis*, *Dialister hominis*, and *Oscillibacter* sp*. PEA192* were also associated with perceived stress and state and/or trait anxiety, suggesting partial convergence between condition-related microbial differences and self-reported stress-related psychological measures.

**Table 4:** Nominally significant taxa associated with stress condition (Model 1).

Taxon	Direction (Stress vs Non-stress)	β (SE)	p	N	q
*Dialister massiliensis*	↑ Stress	0.52 (0.10)	0.004**	15	0.567
*Lawsonibacter asaccharolyticus*	↑ Stress	0.59 (0.18)	0.005**	79	0.567
*Duodenibacillus massiliensis*	↓ Stress	−0.61 (0.21)	0.009**	28	0.621
*Dialister hominis*	↑ Stress	0.48 (0.11)	0.010**	13	0.621
*Sutterella wadsworthensis*	↓ Stress	−0.34 (0.15)	0.018*	59	0.847
*Oscillibacter hominis*	↑ Stress	0.27 (0.10)	0.020*	79	0.847
*Oscillibacter sp. PEA192*	↑ Stress	0.28 (0.10)	0.024*	79	0.856
*Phocaeicola coprophilus*	↓ Stress	−0.22 (0.11)	0.032*	76	0.915
*Blautia obeum*	↓ Stress	−0.23 (0.11)	0.033*	79	0.915
*Phascolarctobacterium sp. Marseille-Q4147*	↓ Stress	−0.12 (0.07)	0.037*	72	0.915

Notes. Tables summarize nominally significant (p < .05) results from exploratory MaAsLin3 mixed-effects models fitted to centred log-ratio–transformed species-level relative abundance data, with a random intercept for participant ID. N reflects the number of samples with non-zero abundance for each taxon. Direction arrows indicate higher (↑) or lower (↓) relative abundance with higher values of the predictor. False discovery rate–adjusted q-values are reported for transparency. p < .05*, p < .01**, p < .001***.

### 4.7 Differential abundance associated with CAR reactivity

When cortisol reactivity (AUCi) was included as a covariate (Model 2), several stress-associated taxa identified in Model 1 remained nominally significant, including *Dialister massiliensis*, *Lawsonibacter asaccharolyticus*, *Dialister hominis*, and *Oscillibacter* sp*.* PEA192 (Table 5). In addition, *Blautia hansenii* and *Enterocloster bolteae* emerged as nominally associated with stress after adjustment for AUCi.

**Table 5:** Nominally significant taxa associated with stress condition, adjusted for cortisol reactivity (AUCi) (Model 2).

Taxon	Direction (Stress vs Non-stress)	β (SE)	p	N	q
*Dialister massiliensis*	↑ Stress	0.49 (0.11)	0.009**	15	1.000
*Ruminococcus champanellensis*	↓ Stress	−0.40 (0.16)	0.014*	70	1.000
*Dialister hominis*	↑ Stress	0.48 (0.12)	0.020*	13	1.000
*Bifidobacterium pseudocatenulatum*	↑ Stress	0.41 (0.16)	0.023*	74	1.000
*Culoivirus americanus*	↓ Stress	−0.98 (0.37)	0.024*	25	1.000
*Lawsonibacter asaccharolyticus*	↑ Stress	0.51 (0.21)	0.027*	79	1.000
*Oscillibacter sp. PEA192*	↑ Stress	0.28 (0.12)	0.038*	79	1.000
*Phascolarctobacterium sp. Marseille-Q4147*	↓ Stress	−0.14 (0.08)	0.044*	72	1.000
*Blautia hansenii*	↑ Stress	0.43 (0.20)	0.048*	78	1.000
*Enterocloster bolteae*	↑ Stress	0.32 (0.14)	0.049*	79	1.000

Notes. Tables summarize nominally significant (p < .05) results from exploratory MaAsLin3 mixed-effects models fitted to centred log-ratio–transformed species-level relative abundance data, with a random intercept for participant ID. N reflects the number of samples with non-zero abundance for each taxon. Direction arrows indicate higher (↑) or lower (↓) relative abundance with higher values of the predictor. False discovery rate–adjusted q-values are reported for transparency. p < .05*, p < .01**, p < .001***.

Independent of stress condition, AUCi was associated with the relative abundance of several taxa. Higher cortisol reactivity was positively associated with *Dabirmavirus hominis*, *Culoivirus americanus*, *Ruminococcus bicirculans*, *Roseburia hominis*, and *Blautia hydrogenotrophica*, whereas *Methanobrevibacter smithii* was negatively associated with AUCi (Table 6).

**Table 6:** Nominally significant taxa associated with cortisol reactivity (AUCi), adjusted for stress condition (Model 2).

Taxon	Direction	β (SE)	p	N	q
*Dabirmavirus hominis*	↑ AUCi	1.63 (0.16)	< 0.001***	12	0.267
*Methanobrevibacter smithii*	↓ AUCi	−1.52 (0.35)	0.001**	18	0.271
*Culoivirus americanus*	↑ AUCi	0.53 (0.20)	0.023*	25	1.000
*Ruminococcus bicirculans*	↑ AUCi	0.65 (0.30)	0.032*	78	1.000
*Roseburia hominis*	↑ AUCi	0.30 (0.14)	0.035*	79	1.000
*Blautia hydrogenotrophica*	↑ AUCi	0.24 (0.12)	0.038*	78	1.000

Note. Table reports nominal p < .05 associations from exploratory MaAsLin3 mixed-effects models using the externally filtered Bracken count matrix and a participant-specific random intercept. CLR normalization was performed internally by MaAsLin3 (transform = NONE); raw AUCi was standardised internally in AUCi-containing models. N denotes MaAsLin3's feature-level number of non-zero observations and is not the overall model sample size. Benjamini–Hochberg FDR-adjusted q-values are shown; q < 0.05 defines evidence surviving multiple-testing correction.

### 4.8 Cortisol reactivity as a moderator of stress-related microbial abundance

Model 3 was interpreted primarily as a test of the Stress Condition × AUCi interaction; the stress-condition and AUCi coefficients reported in Table 7, Table 8 are conditional main effects and are presented for completeness. Findings were broadly consistent in the interaction model, although *Alistipes* sp. i18-0019-D1 emerged as an additional taxon negatively associated with AUCi. (Table 7, Table 8, Table 9). Exploratory interaction analyses further identified nominal stress × cortisol reactivity effects for a subset of taxa (Table 9). Specifically, negative interaction terms were observed for *Oscillibacter hominis*, *Eubacterium ramulus*, and *Oscillibacter* sp. PEA192, indicating that stress-related differences in abundance for these taxa were attenuated at higher levels of cortisol reactivity.

**Table 7:** Nominally significant taxa associated with Stress condition in the Stress × AUCi model (Model 3).

Taxon	Direction (Stress vs Non-stress)	β (SE)	p	N	q
*Ruminococcus champanellensis*	↓ Stress	−0.41 (0.16)	0.014*	70	1.000
*Dialister massiliensis*	↑ Stress	0.48 (0.12)	0.024*	15	1.000
*Bifidobacterium pseudocatenulatum*	↑ Stress	0.41 (0.16)	0.024*	74	1.000
*Lawsonibacter asaccharolyticus*	↑ Stress	0.50 (0.21)	0.031*	79	1.000
*Phascolarctobacterium sp. Marseille-Q4147*	↓ Stress	−0.15 (0.08)	0.040*	72	1.000
*Oscillibacter sp. PEA192*	↑ Stress	0.27 (0.11)	0.040*	79	1.000
*Culoivirus americanus*	↓ Stress	−0.83 (0.36)	0.045*	25	1.000
*Duodenibacillus massiliensis*	↓ Stress	−0.50 (0.23)	0.048*	28	1.000
*Dialister hominis*	↑ Stress	0.46 (0.14)	0.049*	13	1.000

Notes. Tables summarize nominally significant (p < .05) results from exploratory MaAsLin3 mixed-effects models fitted to centred log-ratio–transformed species-level relative abundance data, with a random intercept for participant ID. N reflects the number of samples with non-zero abundance for each taxon. Direction arrows indicate higher (↑) or lower (↓) relative abundance with higher values of the predictor. False discovery rate–adjusted q-values are reported for transparency. p < .05*, p < .01**, p < .001***.

**Table 8:** Nominally significant taxa associated with cortisol reactivity (AUCi) in the Stress × AUCi model (Model 3).

Taxon	Direction	β (SE)	p	N	q
*Methanobrevibacter smithii*	↓ AUCi	−1.53 (0.41)	0.004**	18	1.000
*Blautia hydrogenotrophica*	↑ AUCi	0.40 (0.17)	0.023*	78	1.000
*Bifidobacterium pseudocatenulatum*	↓ AUCi	−0.40 (0.17)	0.027*	74	1.000
*Alistipes sp. i18-0019-D1*	↓ AUCi	−0.26 (0.11)	0.028*	77	1.000
*Dabirmavirus hominis*	↑ AUCi	1.41 (0.35)	0.030*	12	1.000
*Duodenibacillus massiliensis*	↓ AUCi	−0.67 (0.31)	0.048*	28	1.000

Notes. Tables summarize nominally significant (p < .05) results from exploratory MaAsLin3 mixed-effects models fitted to centred log-ratio–transformed species-level relative abundance data, with a random intercept for participant ID. N reflects the number of samples with non-zero abundance for each taxon. Direction arrows indicate higher (↑) or lower (↓) relative abundance with higher values of the predictor. False discovery rate–adjusted q-values are reported for transparency. p < .05*, p < .01**, p < .001***.

**Table 9:** Nominal stress × cortisol reactivity (AUCi) interaction effects (Model 3).

Taxon	Interaction Interpretation	β (SE)	p	N	q
*Oscillibacter hominis*	Stress-related difference decreases with higher AUCi	−0.26 (0.12)	0.025*	79	1.000
*Eubacterium ramulus*	Stress-related difference decreases with higher AUCi	−0.29 (0.14)	0.041*	79	1.000
*Oscillibacter sp. PEA192*	Stress-related difference decreases with higher AUCi	−0.24 (0.13)	0.049*	79	1.000

Notes. Tables summarize nominally significant (p < .05) results from exploratory MaAsLin3 mixed-effects models fitted to centred log-ratio–transformed species-level relative abundance data, with a random intercept for participant ID. N reflects the number of samples with non-zero abundance for each taxon. Direction arrows indicate higher (↑) or lower (↓) relative abundance with higher values of the predictor. False discovery rate–adjusted q-values are reported for transparency. p < .05*, p < .01**, p < .001***.

Within-subject associations between change in cortisol reactivity and change in species abundance.

Exploratory within-subject delta analyses identified 12 species-level associations at nominal p < .05 (Table 10). Positive associations were observed for *Dabirmavirus hominis*, *Phascolarctobacterium succinatutens*, *Simiaoa sunii*, *Phocaeicola vulgatus*, *Bacteroides finegoldii*, *Catenibacterium sp. co_0103*, *Pseudoflavonifractor gallinarum*, *Faecalibacillus intestinalis*, *Bacteroides stercoris*, and *Roseburia hominis*, whereas *Haemophilus parainfluenzae* and *Coprococcus catus* showed negative associations. Only *Dabirmavirus hominis* survived Benjamini–Hochberg FDR correction (q = 0.046); the remaining associations were nominal and are interpreted as exploratory. *Dabirmavirus hominis* and *Roseburia hominis* were the only taxa also identified in the repeated-measures AUCi models, suggesting that the change-score analysis captured partly distinct within-person CLR variation.

**Table 10:** Nominally significant abundance features associated with within-subject change in cortisol reactivity.

Taxon	Direction	B (SE)	p	N	q
*Dabirmavirus hominis*	↑	1.03 (0.21)	< 0.001***	19	0.046
*Haemophilus parainfluenzae*	↓	−0.98 (0.29)	0.006**	17	0.548
*Coprococcus catus*	↓	−0.30 (0.08)	0.007**	16	0.548
*Phascolarctobacterium succinatutens*	↑	1.03 (0.34)	0.008**	17	0.548
*Simiaoa sunii*	↑	0.29 (0.10)	0.009**	16	0.548
*Phocaeicola vulgatus*	↑	0.16 (0.06)	0.009**	21	0.548
*Bacteroides finegoldii*	↑	0.17 (0.08)	0.020*	24	0.871
*Catenibacterium* sp. co_0103	↑	0.85 (0.38)	0.036*	17	0.942
*Pseudoflavonifractor gallinarum*	↑	0.26 (0.13)	0.038*	22	0.942
*Faecalibacillus intestinalis*	↑	1.24 (0.55)	0.039*	14	0.942
*Bacteroides stercoris*	↑	0.53 (0.25)	0.040*	17	0.942
*Roseburia hominis*	↑	0.34 (0.16)	0.043*	17	0.942

**Note.** Table reports nominally significant (*p* < .05) associations from the exploratory within-subject change model. Participant-level ΔCLR values were calculated as Stress − Non-stress after addition of a 0.5 pseudocount to the externally filtered count matrix and sample-wise CLR transformation. Model 4 was fitted to participant-level change scores and therefore did not include a participant random intercept. ΔCLR represents change in a taxon's log-ratio position relative to the geometric mean of the retained community rather than change in absolute abundance. N denotes the feature-level number of non-zero ΔCLR observations reported by MaAsLin3. Benjamini–Hochberg FDR-adjusted q-values are shown; q < 0.05 indicates an association surviving multiple-testing correction. p < .05*, p < .01**, p < .001***.

### 4.9 Sensitivity analyses

Species-level sensitivity analyses supported the directional stability of the primary stress-condition coefficients (Table S17). Across the complete-pair, age-, sex-, and BMI-adjusted, and sleep-, sedentary-, vegetable-, and combined-lifestyle-adjusted specifications, all 10 primary coefficients retained their original direction, although some estimates were attenuated in the more highly adjusted models. Sex-stratified estimates were also largely directionally consistent, and female-specific estimates were generally similar after additional adjustment for hormonal contraceptive use where estimable (Table S17).

## 5 Discussion

The present study examined associations between examination stress, CAR reactivity, and gut microbiome structure using a repeated-measures design. Examination stress was associated with clear psychological and behavioural changes, such as increased perceived stress, anxiety, negative affect, poorer subjective sleep, as well as endocrinological change in terms of amplified CAR. These changes were not accompanied by global restructuring of the adult gut microbiome. Rather, exploratory analyses indicated selective species-level associations with stress condition, cortisol reactivity, their interaction, and within-subject change in cortisol reactivity. Together, these findings suggest that examination-related stress in healthy adults may be reflected more readily in targeted microbial variation than in broad disruption of community structure.

The stress manipulation reliably differentiated stress and non-stress periods, consistent with prior examination-stress research (Kunz-Ebrecht et al., 2004; Powell and Schlotz, 2012). Examination stress was associated with marked psychological changes and altered HPA-axis dynamics, characterised by amplified post-awakening cortisol reactivity despite comparable cortisol concentrations at awakening. This pattern indicates heightened reactive mobilisation rather than elevated basal morning cortisol, consistent with context-dependent modulation of the CAR (Clow et al., 2010; Fries et al., 2009; Stalder et al., 2016).

Despite these psychological and endocrine differences, global microbiome structure remained stable. Alpha diversity did not differ between conditions, beta diversity analyses showed substantial overlap in community composition with no evidence of altered multivariate dispersion, and cortisol reactivity was unrelated to global diversity or community-level change. This pattern is consistent with evidence that adult gut microbial communities are relatively stable over weeks to months, with larger community shifts more typically observed after stronger perturbations such as antibiotic exposure or marked dietary change (David et al., 2014; Dethlefsen and Relman, 2011; Faith et al., 2013; Falony et al., 2016; Lozupone et al., 2012). Given this heterogeneous pattern, considering both global diversity metrics and taxon-level differences is informative because it allows broad community-level stability to be distinguished from more selective microbial associations.

Exploratory species-level analyses identified selective associations with stress condition, cortisol reactivity, and their interaction. In the primary stress-condition model, examination stress was associated with higher abundance of *Dialister massiliensis*, *Dialister hominis*, *Lawsonibacter asaccharolyticus*, *Oscillibacter hominis*, and *Oscillibacter* sp. PEA192, and lower abundance of *Duodenibacillus massiliensis*, *Sutterella wadsworthensis*, *Phocaeicola coprophilus*, *Blautia obeum*, and *Phascolarctobacterium* sp. Marseille-Q4147. Several taxa identified in the primary condition model, including *Duodenibacillus massiliensis*, *Lawsonibacter asaccharolyticus*, *Phascolarctobacterium* sp. Marseille-Q4147, *Sutterella wadsworthensis*, *Dialister hominis*, and *Oscillibacter* sp. PEA192, also appeared in supplemental models of perceived stress and anxiety, further supporting the interpretation that the stress condition captured meaningful stress-related microbial variation. These differences occurred without reduced alpha diversity or increased multivariate dispersion, supporting selective taxon-level variation rather than generalised dysbiosis. This pattern aligns with Ma et al. (2023), who reported heterogeneous associations between psychological stress and global microbiome diversity but more recurrent associations at the taxonomic level, including reduced *Sutterella* and *Phascolarctobacterium* under higher stress.

Independent of stress condition, AUCi was associated with a largely distinct set of taxa. Positive associations were observed with *Dabirmavirus hominis*, *Culoivirus americanus*, *Ruminococcus bicirculans*, *Roseburia hominis*, and *Blautia hydrogenotrophica*, whereas *Methanobrevibacter smithii* showed a negative association. The limited overlap between stress-condition and AUCi-associated taxa suggests that cortisol reactivity captured a distinct physiological dimension of stress–microbiome variation beyond examination exposure itself. This interpretation was further supported by the interaction model, in which examination-related abundance differences in *Oscillibacter hominis*, *Oscillibacter* sp. PEA192, and *Eubacterium ramulus* were attenuated at higher AUCi, indicating that physiological stress responsivity may moderate how contextual stress is reflected in gut microbial composition.

Within-subject change analyses provided additional exploratory evidence that changes in cortisol reactivity covaried with changes in species abundance. Greater increases in AUCi were associated with increased abundance of several taxa, including *Dabirmavirus hominis*, *Phascolarctobacterium succinatutens*, *Phocaeicola vulgatus*, *Bacteroides finegoldii*, *Bacteroides stercoris*, and *Roseburia hominis*, whereas *Haemophilus parainfluenzae* and *Coprococcus catus* showed the opposite pattern. Only *Dabirmavirus hominis* and *Roseburia hominis* overlapped with the repeated-measures AUCi models, suggesting that change-score analyses captured partly distinct within-person microbial variation. Within a bidirectional microbiota–gut–brain axis framework, this pattern is consistent with neuroendocrine state and the intestinal microbial environment varying together across stress contexts, without implying that cortisol changes directly caused the observed microbial shifts or vice versa (Cryan et al., 2019; Rusch et al., 2023). Importantly, these change-score estimates are based on a single stool sample per condition and therefore have limited precision for separating stress-related change from ordinary day-to-day intraindividual microbiome variation. Furthermore, ΔCLR denotes change relative to the geometric mean of the retained community rather than absolute microbial abundance. Only the Dabirmavirus hominis association survived FDR correction; the remaining change-score associations should therefore be regarded as nominal exploratory signals.

Several taxa associated with stress condition, AUCi, and within-subject change have been implicated in fermentative or SCFA-linked ecological processes, suggesting that stress-related microbial variation may partly involve substrate degradation, succinate utilisation, short-chain fatty acid production, and microbial cross-feeding pathways relevant to host stress physiology. Among stress-condition taxa, *Oscillibacter* species are strict anaerobes, with related species characterised by production of valerate, a fermentation-derived short-chain fatty acid (Iino et al., 2007; Louis and Flint, 2017). Notably, *Oscillibacter hominis* and *Oscillibacter* sp. PEA192 were also identified in the interaction model, where examination-related abundance differences were attenuated at higher AUCi, suggesting that these taxa may index the combined influence of stress context and physiological stress responsivity. *Phascolarctobacterium* species utilise succinate and contribute to propionate production in the colon (Louis and Flint, 2017), with *P. succinatutens* specifically characterised as a succinate-utilising species isolated from human faeces (Yohei et al., 2012). *Dialister* species have also been identified as major succinate consumers in the human gut and may reflect shifts in succinate-linked fermentative ecology (Anthamatten et al., 2024).

Among AUCi-associated taxa, *Roseburia hominis* is a recognised butyrate-producing species (Louis and Flint, 2017; Machiels et al., 2014), with preclinical evidence linking its neuroimmune effects to SCFA-mediated histone deacetylase inhibition (Song et al., 2022). *Ruminococcus bicirculans* is a plant-glucan degrader that may contribute upstream to SCFA-producing networks through fibre breakdown and substrate provision (Louis and Flint, 2017; Wegmann et al., 2014). Several taxa identified in the within-subject change analyses are also linked to SCFA-related fermentative metabolism, including the butyrate-associated *Coprococcus catus* (Notting et al., 2023; Reichardt et al., 2014), the succinate-utilising and propionate-producing *Phascolarctobacterium succinatutens* (Anthamatten et al., 2024; Yohei et al., 2012), and *Phocaeicola vulgatus*, which produces organic acids including acetate, succinate, and lactate under anaerobic conditions (Keitel et al., 2025).

Short-chain fatty acids are increasingly regarded as a mechanistic interface within the microbiota–gut–brain axis, linking microbial metabolism to host stress physiology through effects on epithelial barrier integrity, immune signalling, vagal afferent pathways, and neuroendocrine regulation (Cryan et al., 2019; Dalile et al., 2019; Parada Venegas et al., 2019). Preclinical evidence indicates that SCFA supplementation can attenuate stress-induced brain–gut axis alterations, including effects on corticosterone signalling and stress-related behaviour (van de Wouw et al., 2018), while human experimental evidence suggests that colonic SCFA delivery can attenuate cortisol responses to psychosocial stress (Dalile et al., 2020). This interpretation is consistent with Karner et al. (2026), who found that inferred butyrate- and propionate-producing capacity were differentially associated with salivary cortisol reactivity to acute stress in healthy adults. Specifically, greater inferred butyrate-producing capacity was associated with higher cortisol stress reactivity, whereas greater inferred propionate-producing capacity was associated with lower cortisol stress reactivity, suggesting that SCFA-linked microbial features may relate to host stress signalling in a metabolite-specific manner.

Together, these findings suggest that SCFA-linked microbial pathways may provide a plausible bottom-up mechanism through which gut microbial variation relates to host stress reactivity and neuroendocrine signalling. However, because these interpretations are inferred from taxonomic associations rather than direct pathway or metabolite measurements, they should be regarded as provisional and hypothesis-generating. Replication in larger samples with direct functional profiling, including metagenomic pathway analysis and metabolomics, will be required to determine whether cortisol-linked taxonomic patterns correspond to meaningful differences in SCFA metabolism and host–microbe signalling. Taxonomic abundance alone does not establish the abundance, expression, or activity of the metabolic pathways encoded by a microbial community. Accordingly, the SCFA-related interpretation should be regarded as a plausible mechanistic hypothesis rather than evidence of altered pathway activity or SCFA production.

### 5.1 Methodological considerations and limitations

Several limitations warrant acknowledgment. First, the study was not powered to detect small taxa-level effects after correction for multiple testing, a common constraint in human microbiome research characterised by high dimensionality, correlated features, and modest psychosocial effect sizes (Falony et al., 2016; Knight et al., 2018; Yang and Chen, 2022). Accordingly, species-level associations should be interpreted as exploratory and hypothesis-generating rather than confirmatory. Second, CAR assessment relied on participant adherence to prescribed sampling schedules without objective verification through actigraphy or electronic timestamping. Nonetheless, cortisol concentrations showed the expected post-awakening rise in both conditions and a stronger response during the examination period, supporting the validity of the CAR assessment. Although protocol adherence was high, minor timing deviations cannot be excluded and may have attenuated associations involving CAR indices (Stalder et al., 2016).

Third, dietary intake was assessed using the Leeds Short Food Frequency Questionnaire to minimise participant burden and confirm broad dietary stability across conditions (Cleghorn et al., 2016). Although suitable for habitual dietary patterns, this measure may have lacked sensitivity to short-term microbiome-relevant changes, such as meal timing, fermentable substrate intake, or caffeine consumption.

Behavioural measures also differed between conditions, with poorer subjective sleep quality, lower vegetable intake, and greater sedentary time during the examination period. These changes represent plausible alternative explanations for some of the observed microbiome associations, although they may also form part of the behavioural response to examination stress and contribute to stress-related microbial variation, rather than acting solely as conventional baseline confounders. Sensitivity analyses were therefore used to assess the robustness of the primary species-level findings to adjustment for these factors, with emphasis placed on the stability of coefficient direction and magnitude across model specifications rather than on whether nominal significance was retained after additional covariate adjustment.

The microbiome sampling design introduces additional sources of uncertainty. A single stool sample was collected during each assessment condition, and repeated sampling within each assessment window may have provided a more precise estimate of condition-specific microbiome composition by reducing the influence of short-term intraindividual variability (Johnson et al., 2020). Exact stool collection time was not recorded or standardised, preventing assessment of potential diurnal variation, while examination clock time and overall examination workload were also unavailable. Examination-format comparisons were additionally constrained by the small and heterogeneous non-written subgroup. Although all samples were sequenced within a single sequencing run using a consistent downstream bioinformatic workflow, residual biological, sampling, and technical variability cannot be excluded. Importantly, CLR transformation addresses the compositional nature of microbiome data but does not remove these sources of variability. These considerations are particularly relevant to the exploratory within-subject ΔCLR analysis, in which differences between two single time-point samples may reflect both stress-related variation and ordinary short-term fluctuations in microbial composition.

### 5.2 Future directions

Future work should better distinguish between specific stress domains, as examination stress combines anticipatory, cognitive, and psychosocial components that cannot be disentangled in the present design. Controlled laboratory paradigms would provide greater precision over acute stress exposure, while larger longitudinal studies of chronic or cumulative stress would clarify how different stressor types engage neuroendocrine pathways and shape gut microbial ecology over time. Future longitudinal designs would also benefit from including a post-stress recovery phase, in which microbiome and cortisol measures are reassessed after examination demands and CAR responses have returned to baseline. This would help determine whether stress-associated microbial differences are transient, reversible, or maintained beyond the acute stress context.

Direct functional and quantitative profiling is also needed to extend beyond taxonomic inference. Metatranscriptomics, metagenomic pathway analysis, metabolomics, and direct short-chain fatty acid quantification would help determine whether the observed taxa-level associations correspond to meaningful differences in microbial metabolic activity. In addition, because the present microbiome analyses were based on compositional relative abundance data, targeted quantification of individual species using approaches such as quantitative polymerase chain reaction, digital polymerase chain reaction, or spike-in-based absolute abundance estimation would help determine whether the observed associations reflect changes in absolute microbial abundance. These approaches would allow direct interrogation of pathways implicated here, including short-chain fatty acid production, polyphenol metabolism, and immune modulation, thereby strengthening mechanistic inference regarding microbiota–gut–brain axis processes under stress.

## 6 Conclusion

The present findings indicate that stress exposure and physiological stress responsivity represent dissociable dimensions of stress–microbiome association. Transitions between stress and non-stress conditions were not accompanied by changes in global microbial diversity or community structure, consistent with resilience of the adult gut microbiome to transient psychological stress. However, stress condition, CAR reactivity, and within-person change in CAR reactivity were each associated with selective species-level microbial variation, indicating that stress-related biological differences may be reflected in targeted ecological shifts rather than broad community disruption.

By integrating repeated within-subject assessment, whole-metagenome shotgun sequencing, and cortisol-based assessment of HPA-axis responsivity, the present study extends existing evidence beyond exposure-based models of stress. The findings support a microbiota–gut–brain axis framework characterised by community-level stability alongside selective, host-associated ecological variation, and suggest that inter-individual and within-individual differences in neuroendocrine responsivity may contribute to how psychological stress is associated with gut microbial composition. Further longitudinal and mechanistic studies are needed to clarify the pathways through which stress-related neuroendocrine processes and gut microbial ecology vary together across changing contexts.

## CRediT authorship contribution statement

**Connor B. O'Reilly:** Writing – original draft, Visualization, Validation, Project administration, Methodology, Investigation, Formal analysis, Data curation, Conceptualization. **Timo Giesbrecht:** Writing – review & editing, Supervision, Methodology, Funding acquisition, Conceptualization. **John Tyson-Carr:** Writing – review & editing, Supervision, Conceptualization. **Barry Murphy:** Writing – review & editing, Supervision, Methodology, Conceptualization. **Kendall Cagney:** Writing – review & editing, Resources, Methodology, Investigation. **Luca Lenzi:** Validation, Software, Methodology, Formal analysis. **Nicholas Fallon:** Writing – review & editing, Supervision, Methodology, Conceptualization. **Carl A. Roberts:** Writing – review & editing, Supervision, Methodology, Funding acquisition, Conceptualization.

## Ethical approval

The study was approved by the University of Liverpool Central University Research Ethics Committee D (ethics approval number: 13091).

## Declaration of generative AI use

ChatGPT (OpenAI; GPT-5.5) was used during manuscript preparation to assist with language editing, text refinement, formatting, and code debugging. Generative AI tools were not used to generate study data, conduct statistical analyses, interpret results, produce figures or images, or formulate the scientific conclusions presented in this manuscript. The authors reviewed and verified all AI-assisted outputs and take full responsibility for the integrity, accuracy, and originality of the manuscript, including the accuracy of all references.

## Funding

This work was supported by a UKRI BBSRC iCASE studentship with industrial partner Unilever (grant number: BB/X511444/1).

## Declaration of competing interest

TG and BM work for Unilever. Unilever produces beauty and personal care products. However, no Unilever products were used in this study, and no data were collected from participants as to their use, or not, of Unilever products.

## Data Availability

The data and supporting materials associated with this study have been deposited on Zenodo and are available at https://zenodo.org/records/22099709. The repository includes the analysis data, reproducible R scripts, R session and package information, and complete outputs from the primary, supplementary, and sensitivity analyses.
