# Supplementary material for: Not all stress is equal: Examination stress and cortisol reactivity are associated with exploratory species-level variation but not broad restructuring of the gut microbiome
Source: Neurobiol Stress. 2026 Sep 28;45:100855. doi: 10.1016/j.ynstr.2026.100855 (PMC13658120; doi:10.1016/j.ynstr.2026.100855)
Supplement: Supplementary file 1 [file mmc1.docx]

**Supplemental Materials**

**Table S1**

*Condition × Sex Sensitivity Analyses for Subjective Psychological Outcomes*

| **Outcome** | **Female Δ (SE)** | ***p*** | **Male Δ (SE)** | ***p*** | **F(1, 37)** | ***p*** | **ηp²** |
| --- | --- | --- | --- | --- | --- | --- | --- |
| Perceived stress | 4.96 (1.31) | < .001 | 6.25 (1.57) | < .001 | 0.40 | .531 | .011 |
| State anxiety | 11.70 (2.49) | < .001 | 11.56 (2.99) | < .001 | 0.00 | .973 | < .001 |
| Trait anxiety | 5.78 (1.83) | .003 | 8.25 (2.19) | < .001 | 0.75 | .393 | .020 |
| Positive affect | -5.91 (1.43) | < .001 | -3.44 (1.71) | .052 | 1.24 | .274 | .032 |
| Negative affect | 7.87 (1.68) | < .001 | 7.44 (2.02) | < .001 | 0.03 | .870 | < .001 |
| Self-esteem | 0.83 (0.58) | .161 | 0.56 (0.69) | .421 | 0.09 | .771 | .002 |

*Note.* Female n = 23; male n = 16. Δ represents the estimated within-sex condition difference (Stress − Non-stress); positive values indicate higher scores during the examination-stress condition. Within-sex contrasts were estimated from the Condition × Sex mixed models. The F, p, and partial eta-squared (ηp²) columns report the Condition × Sex interaction. No interaction was statistically significant.

| ***Table S2. Summary statistics and paired differences for subjective sleep measures across stress and non-stress conditions*** | | | | | | | | | |
| --- | --- | --- | --- | --- | --- | --- | --- | --- | --- |
| ***Variable*** | ***Condition*** | ***N*** | ***Mean*** | ***SD*** | ***Paired Difference (Stress − Non-stress)*** | ***SD*** | ***95% CI*** | ***p*** |  |
| Subjective Sleep Quality | Non-stress | 39 | 0.90 | 0.64 | 0.54 | 0.64 | [0.33, 0.75] | < .001*** |  |
|  | Stress | 39 | 1.44 | 0.72 |  |  |  |  |  |
| Sleep Latency | Non-stress | 39 | 1.44 | 0.50 | 0.15 | 0.67 | [−0.06, 0.37] | .08 |  |
|  | Stress | 39 | 1.59 | 0.50 |  |  |  |  |  |
| Sleep Disturbances | Non-stress | 39 | 1.21 | 0.41 | 0.26 | 0.55 | [0.08, 0.43] | .003** |  |
|  | Stress | 39 | 1.46 | 0.55 |  |  |  |  |  |
| Use of Sleep Medication | Non-stress | 39 | 0.05 | 0.32 | 0.03 | 0.16 | [−0.03, 0.08] | .16 |  |
|  | Stress | 39 | 0.08 | 0.35 |  |  |  |  |  |
| Daytime Dysfunction | Non-stress | 39 | 2.10 | 0.31 | −0.03 | 0.28 | [−0.12, 0.07] | .29 |  |
|  | Stress | 39 | 2.08 | 0.27 |  |  |  |  |  |
| Habitual Sleep Efficiency | Non-stress | 39 | 0.54 | 0.68 | 0.18 | 0.76 | [−0.07, 0.42] | .07 |  |
|  | Stress | 39 | 0.72 | 0.79 |  |  |  |  |  |
| Sleep Duration | Non-stress | 39 | 0.26 | 0.44 | 0.23 | 0.58 | [0.04, 0.42] | .01* |  |
|  | Stress | 39 | 0.49 | 0.60 |  |  |  |  |  |
| Global Sleep Score | Non-stress | 39 | 7.03 | 2.06 | 1.67 | 2.68 | [0.80, 2.54] | < .001*** |  |
|  | Stress | 39 | 8.69 | 2.68 |  |  |  |  |  |
| **Notes.** Values are presented as *M* ± *SD*. Paired differences are calculated as Stress − Non-stress condition scores. Statistical comparisons were conducted using paired-samples *t*-tests (two-tailed). Confidence intervals reflect 95% confidence levels. Higher scores indicate poorer sleep across all Pittsburgh Sleep Quality Index (PSQI) component scores and the global PSQI score. *p* < .05*, *p* < .01**, *p* < .001***. | | | | | | | | | |

Table S3. ***Summary statistics and paired differences for self-reported dietary quality across stress and non-stress conditions***

| ***Variable*** | ***Condition*** | ***N*** | ***Mean*** | ***SD*** | ***Paired Difference (Stress - Non-stress)*** | ***SD*** | ***95% CI*** | ***p*** |
| --- | --- | --- | --- | --- | --- | --- | --- | --- |
| Fruit | Non-stress | 39 | 2.10 | 0.68 | -0.05 | 0.69 | [-0.27, 0.17] | .32 |
|  | Stress | 39 | 2.05 | 0.65 |  |  |  |  |
| Vegetables | Non-stress | 39 | 2.21 | 0.77 | -0.23 | 0.71 | [-0.46, -0.00] | .02* |
|  | Stress | 39 | 1.97 | 0.81 |  |  |  |  |
| Oily Fish | Non-stress | 39 | 1.69 | 0.69 | 0.03 | 0.67 | [-0.19, 0.24] | .41 |
|  | Stress | 39 | 1.72 | 0.69 |  |  |  |  |
| Fats | Non-stress | 39 | 2.26 | 0.88 | 0.00 | 0.95 | [-0.31, 0.31] | .50 |
|  | Stress | 39 | 2.26 | 0.82 |  |  |  |  |
| Non-milk extrinsic sugars (NMES) | Non-stress | 39 | 2.69 | 0.57 | -0.10 | 0.97 | [-0.42, 0.21] | .26 |
|  | Stress | 39 | 2.59 | 0.79 |  |  |  |  |
| Dietary Quality Score (DQS) | Non-stress | 39 | 10.95 | 1.57 | -0.36 | 1.56 | [-0.87, 0.15] | .08 |
|  | Stress | 39 | 10.59 | 1.27 |  |  |  |  |

*Note.* Values are presented as means and standard deviations. Paired differences were calculated as stress minus non-stress condition scores. Statistical comparisons were conducted using paired-samples *t* tests (two-tailed). Confidence intervals reflect 95% confidence levels. Higher scores indicate greater frequency of consumption for individual food groups and higher overall dietary quality for the Dietary Quality Score (DQS). *p* < .05*, *p* < .01**, *p* < .001***.

***Table S4.*** ***Summary statistics and paired differences for self-reported physical activity across stress and non-stress conditions***

| ***Variable*** | ***Condition*** | ***N*** | ***M*** | ***SD*** | ***Paired difference (Stress − Non-stress)*** | ***SD*** | ***95% CI*** | ***p*** |
| --- | --- | --- | --- | --- | --- | --- | --- | --- |
| Total physical activity (min/week) | Non-stress | 34 | 878.97 | 909.49 | −96.62 | 979.22 | [−410.01, 255.06] | .19 |
|  | Stress | 34 | 782.35 | 841.61 |  |  |  |  |
| Moderate-to-vigorous physical activity (MVPA; min/week) | Non-stress | 30 | 631.33 | 692.90 | −90.50 | 905.87 | [−382.17, 267.50] | .05 |
|  | Stress | 30 | 540.83 | 761.54 |  |  |  |  |
| Metabolic equivalent tasks (MET-min/week) | Non-stress | 34 | 4622.90 | 5075.00 | −428.82 | 5777.60 | [−2320.07, 1651.54] | .22 |
|  | Stress | 34 | 4194.10 | 4908.70 |  |  |  |  |
| Sedentary time (min/day) | Non-stress | 39 | 357.05 | 144.50 | 37.31 | 163.65 | [−17.18, 85.90] | .04* |
|  | Stress | 39 | 394.36 | 158.44 |  |  |  |  |

*Note.* Values are presented as means and standard deviations. Paired differences were calculated as stress minus non-stress condition scores. Statistical comparisons were conducted using two-tailed paired-samples t tests. Confidence intervals are 95% confidence intervals. Sample size varied across variables because of missing or implausible responses identified using a priori plausibility criteria. MVPA = moderate-to-vigorous physical activity; MET = metabolic equivalent task. p < .05*, p < .01**, p < .001***.

Table S5. Sex-Sensitivity Analyses for the Cortisol Awakening Response and Derived Cortisol Indices

**Panel A. Omnibus sex-moderation effects for the cortisol awakening response**

| **Effect** | **df** | **F** | ***p*** | **ηp²** |
| --- | --- | --- | --- | --- |
| Condition × Sex | 1, 33 | 0.22 | .641 | .007 |
| Time × Condition × Sex | 1.71, 56.36 | 2.19 | .129 | .062 |

**Panel B. Condition × Sex analyses for derived cortisol indices**

| **Index** | **Female Δ (SE)** | ***p*** | **Male Δ (SE)** | ***p*** | **F(1, 33)** | ***p*** | **ηp²** |
| --- | --- | --- | --- | --- | --- | --- | --- |
| AUCi | 156.59 (52.15) | .005 | 15.76 (63.87) | .807 | 2.92 | .097 | .081 |
| AUCg | 84.58 (55.48) | .137 | 61.76 (67.95) | .370 | 0.07 | .796 | .002 |
| Peak cortisol | 3.92 (1.65) | .023 | 1.15 (2.02) | .573 | 1.13 | .296 | .033 |
| Maximum increase | 5.53 (1.95) | .008 | 0.72 (2.39) | .765 | 2.43 | .128 | .069 |

*Note.* Female n = 21; male n = 14. Panel A reports the Condition × Sex and Time × Condition × Sex interaction terms from the mixed ANOVA of cortisol concentrations measured at awakening, +30 min, and +45 min. Greenhouse–Geisser-corrected degrees of freedom are shown for the three-way interaction. In Panel B, Δ represents the estimated within-sex condition difference (Stress − Non-stress); positive values indicate higher values during the examination-stress condition. The F, p, and partial eta-squared (ηp²) columns in Panel B report the formal Condition × Sex interaction. AUCi = area under the curve with respect to increase; AUCg = area under the curve with respect to ground. No sex-moderation interaction reached statistical significance.

**Single-predictor mixed-effects models of species-level microbial abundance**

Table S6. Nominally significant taxa associated with AUCi

| **Taxon** | **Direction (Higher vs Lower AUCi)** | **β (SE)** | **p** | **N** | **q** |
| --- | --- | --- | --- | --- | --- |
| Dabirmavirus hominis | ↑ Higher values | 1.57 (0.16) | < .001*** | 12 | .047 |
| Methanobrevibacter smithii | ↓ Higher values | -1.57 (0.36) | < .001*** | 17 | .080 |
| Duodenibacillus massiliensis | ↓ Higher values | -0.76 (0.32) | .027* | 27 | 1.000 |
| Vescimonas coprocola | ↑ Higher values | 0.26 (0.11) | .027* | 74 | 1.000 |
| Roseburia hominis | ↑ Higher values | 0.28 (0.14) | .048* | 74 | 1.000 |

Notes. Tables summarize nominally significant (p < .05) results from exploratory MaAsLin3 mixed-effects models fitted to centred log-ratio–transformed species-level relative abundance data, with a random intercept for participant ID. N reflects the number of samples with non-zero abundance for each taxon. Direction arrows indicate higher (↑) or lower (↓) relative abundance with higher values of the predictor. False discovery rate–adjusted q-values are reported for transparency. p < .05*, p < .01**, p < .001***.

Table S7. Nominally significant taxa associated with AUCg

| **Taxon** | **Direction (Higher vs Lower AUCg)** | **β (SE)** | **p** | **N** | **q** |
| --- | --- | --- | --- | --- | --- |
| uncultured Alistipes sp. | ↓ Higher values | -0.34 (0.13) | .009** | 73 | 1.000 |
| Hungatella hathewayi | ↓ Higher values | -0.29 (0.12) | .013* | 74 | 1.000 |
| Wujia chipingensis | ↓ Higher values | -0.53 (0.21) | .015* | 72 | 1.000 |
| Phascolarctobacterium faecium | ↓ Higher values | -0.61 (0.25) | .021* | 49 | 1.000 |
| Bacteroides fragilis | ↑ Higher values | 0.34 (0.14) | .028* | 74 | 1.000 |
| Methanobrevibacter smithii | ↓ Higher values | -1.54 (0.64) | .028* | 17 | 1.000 |
| Paraprevotella xylaniphila | ↑ Higher values | 0.37 (0.17) | .042* | 73 | 1.000 |

Notes. Tables summarize nominally significant (p < .05) results from exploratory MaAsLin3 mixed-effects models fitted to centred log-ratio–transformed species-level relative abundance data, with a random intercept for participant ID. N reflects the number of samples with non-zero abundance for each taxon. Direction arrows indicate higher (↑) or lower (↓) relative abundance with higher values of the predictor. False discovery rate–adjusted q-values are reported for transparency. p < .05*, p < .01**, p < .001***.

Table S8. Nominally significant taxa associated with perceived stress (PSS)

| **Taxon** | Direction (Higher vs Lower PSS) | **β (SE)** | **p** | **N** | **q** |
| --- | --- | --- | --- | --- | --- |
| Duodenibacillus massiliensis | ↓ Higher values | -0.50 (0.17) | .008** | 28 | .925 |
| Lawsonibacter asaccharolyticus | ↑ Higher values | 0.39 (0.14) | .010** | 79 | .925 |
| Faecalibacillus intestinalis | ↓ Higher values | -0.36 (0.15) | .012* | 78 | .925 |
| Phascolarctobacterium sp. Marseille-Q4147 | ↓ Higher values | -0.16 (0.07) | .015* | 72 | .925 |
| Eubacterium ramulus | ↓ Higher values | -0.18 (0.10) | .035* | 79 | 1.000 |
| Oscillibacter sp. PEA192 | ↑ Higher values | 0.19 (0.08) | .044* | 79 | 1.000 |

Notes. Tables summarize nominally significant (p < .05) results from exploratory MaAsLin3 mixed-effects models fitted to centred log-ratio–transformed species-level relative abundance data, with a random intercept for participant ID. N reflects the number of samples with non-zero abundance for each taxon. False discovery rate–adjusted q-values are reported for transparency. p < .05*, p < .01**, p < .001***.

Table S9. Nominally significant taxa associated with STAI-T

| **Taxon** | **Direction (Higher vs Lower STAI-T)** | **β (SE)** | **p** | **N** | **q** |
| --- | --- | --- | --- | --- | --- |
| Phascolarctobacterium sp. Marseille-Q4147 | ↓ Higher values | -0.23 (0.07) | < .001*** | 72 | .192 |
| Faecalibacillus intestinalis | ↓ Higher values | -0.45 (0.16) | .004** | 78 | .379 |
| Oscillibacter sp. PEA192 | ↑ Higher values | 0.28 (0.08) | .006** | 79 | .379 |
| Methanobrevibacter smithii | ↓ Higher values | -1.25 (0.39) | .008** | 18 | .379 |
| Lawsonibacter asaccharolyticus | ↑ Higher values | 0.43 (0.14) | .009** | 79 | .379 |
| Coprococcus catus | ↓ Higher values | -0.24 (0.10) | .009** | 79 | .379 |
| Duodenibacillus massiliensis | ↓ Higher values | -0.78 (0.29) | .012* | 28 | .417 |
| Flavonifractor plautii | ↑ Higher values | 0.34 (0.12) | .015* | 79 | .467 |
| Sutterella wadsworthensis | ↓ Higher values | -0.34 (0.17) | .030* | 59 | .737 |
| Phascolarctobacterium succinatutens | ↓ Higher values | -0.19 (0.10) | .032* | 68 | .737 |
| Blautia luti | ↓ Higher values | -0.11 (0.07) | .034* | 79 | .737 |
| Agathobaculum massiliense | ↑ Higher values | 0.29 (0.11) | .036* | 77 | .737 |
| Dysosmobacter welbionis | ↑ Higher values | 0.26 (0.10) | .043* | 79 | .781 |
| Faecalibacterium sp. I4-1-79 | ↓ Higher values | -0.27 (0.15) | .046* | 79 | .781 |
| Enterocloster bolteae | ↑ Higher values | 0.27 (0.11) | .048* | 79 | .781 |

Notes. Tables summarize nominally significant (p < .05) results from exploratory MaAsLin3 mixed-effects models fitted to centred log-ratio–transformed species-level relative abundance data, with a random intercept for participant ID. N reflects the number of samples with non-zero abundance for each taxon. Direction arrows indicate higher (↑) or lower (↓) relative abundance with higher values of the predictor. False discovery rate–adjusted q-values are reported for transparency. p < .05*, p < .01**, p < .001***.

Table S10. Nominally significant taxa associated with STAI-S

| **Taxon** | **Direction (Higher vs Lower STAI-S)** | **β (SE)** | **p** | **N** | **q** |
| --- | --- | --- | --- | --- | --- |
| Faecalibacillus intestinalis | ↓ Higher values | -0.39 (0.14) | .002** | 78 | .528 |
| Duodenibacillus massiliensis | ↓ Higher values | -0.69 (0.22) | .004** | 28 | .528 |
| Lawsonibacter asaccharolyticus | ↑ Higher values | 0.41 (0.13) | .007** | 79 | .608 |
| Catenibacterium sp. co 0103 | ↓ Higher values | -0.18 (0.08) | .013* | 74 | .720 |
| Phascolarctobacterium sp. Marseille-Q4147 | ↓ Higher values | -0.12 (0.06) | .014* | 72 | .720 |
| Dialister hominis | ↑ Higher values | 0.40 (0.13) | .018* | 13 | .762 |
| Lachnospira eligens | ↓ Higher values | -0.29 (0.15) | .029* | 79 | .865 |
| Clostridioides difficile | ↓ Higher values | -0.19 (0.10) | .030* | 79 | .865 |
| Coprococcus catus | ↓ Higher values | -0.16 (0.09) | .031* | 79 | .865 |
| Blautia luti | ↓ Higher values | -0.09 (0.06) | .038* | 79 | .956 |
| Sutterella wadsworthensis | ↓ Higher values | -0.24 (0.14) | .047* | 59 | 1.000 |
| Sellimonas catena | ↓ Higher values | -0.09 (0.07) | .050* | 79 | 1.000 |

Notes. Tables summarize nominally significant (p < .05) results from exploratory MaAsLin3 mixed-effects models fitted to centred log-ratio–transformed species-level relative abundance data, with a random intercept for participant ID. N reflects the number of samples with non-zero abundance for each taxon. Direction arrows indicate higher (↑) or lower (↓) relative abundance with higher values of the predictor. False discovery rate–adjusted q-values are reported for transparency. p < .05*, p < .01**, p < .001***.

Table S11. Nominally significant taxa associated with Positive affect

| **Taxon** | **Direction (Higher vs Lower Positive affect)** | **β (SE)** | **p** | **N** | **q** |
| --- | --- | --- | --- | --- | --- |
| Bacteroides uniformis | ↓ Higher values | -0.48 (0.13) | .001** | 79 | .330 |
| Lawsonibacter asaccharolyticus | ↓ Higher values | -0.45 (0.14) | .005** | 79 | .330 |
| Dysosmobacter welbionis | ↓ Higher values | -0.32 (0.10) | .006** | 79 | .330 |
| Oscillibacter sp. PEA192 | ↓ Higher values | -0.27 (0.08) | .007** | 79 | .330 |
| Bacteroides cellulosilyticus | ↓ Higher values | -0.53 (0.17) | .007** | 79 | .330 |
| Bacteroides humanifaecis | ↓ Higher values | -0.35 (0.11) | .009** | 77 | .330 |
| Coprococcus catus | ↑ Higher values | 0.22 (0.10) | .012* | 79 | .330 |
| Phascolarctobacterium sp. Marseille-Q4147 | ↑ Higher values | 0.15 (0.07) | .012* | 72 | .330 |
| Holdemania massiliensis | ↑ Higher values | 0.19 (0.09) | .013* | 79 | .330 |
| Bacteroides faecis | ↓ Higher values | -0.34 (0.11) | .013* | 79 | .330 |
| Parabacteroides goldsteinii | ↓ Higher values | -0.38 (0.13) | .016* | 79 | .359 |
| Coprococcus eutactus | ↑ Higher values | 0.35 (0.16) | .020* | 79 | .379 |
| Eubacterium ramulus | ↑ Higher values | 0.21 (0.10) | .020* | 79 | .379 |
| Blautia luti | ↑ Higher values | 0.12 (0.07) | .021* | 79 | .379 |
| Bacteroides sp. MSB163 | ↓ Higher values | -0.36 (0.13) | .024* | 79 | .398 |
| Bacteroides sp. CACC 737 | ↓ Higher values | -0.36 (0.14) | .027* | 76 | .416 |
| Pusillibacter faecalis | ↓ Higher values | -0.29 (0.11) | .038* | 79 | .560 |

Notes. Tables summarize nominally significant (p < .05) results from exploratory MaAsLin3 mixed-effects models fitted to centred log-ratio–transformed species-level relative abundance data, with a random intercept for participant ID. N reflects the number of samples with non-zero abundance for each taxon. Direction arrows indicate higher (↑) or lower (↓) relative abundance with higher values of the predictor. False discovery rate–adjusted q-values are reported for transparency. p < .05*, p < .01**, p < .001***.

Table S12. Nominally significant taxa associated with Negative affect

| **Taxon** | **Direction (Higher vs Lower Negative affect)** | **β (SE)** | **p** | **N** | **q** |
| --- | --- | --- | --- | --- | --- |
| Duodenibacillus massiliensis | ↓ Higher values | -0.74 (0.19) | .001** | 28 | .329 |
| Phascolarctobacterium sp. Marseille-Q4147 | ↓ Higher values | -0.12 (0.05) | .007** | 72 | .816 |
| Lawsonibacter asaccharolyticus | ↑ Higher values | 0.35 (0.12) | .011* | 79 | .816 |
| Faecalibacillus intestinalis | ↓ Higher values | -0.31 (0.13) | .015* | 78 | .816 |
| Megamonas funiformis | ↓ Higher values | -1.15 (0.34) | .016* | 14 | .816 |
| Eubacterium ventriosum | ↓ Higher values | -0.27 (0.13) | .037* | 79 | 1.000 |
| Enterocloster bolteae | ↑ Higher values | 0.21 (0.08) | .041* | 79 | 1.000 |

Notes. Tables summarize nominally significant (p < .05) results from exploratory MaAsLin3 mixed-effects models fitted to centred log-ratio–transformed species-level relative abundance data, with a random intercept for participant ID. N reflects the number of samples with non-zero abundance for each taxon. Direction arrows indicate higher (↑) or lower (↓) relative abundance with higher values of the predictor. False discovery rate–adjusted q-values are reported for transparency. p < .05*, p < .01**, p < .001***.

Table S13. Nominally significant taxa associated with Rosenberg Self-esteem Scale

| **Taxon** | **Direction (Higher vs Lower Rosenberg self-esteem)** | **β (SE)** | **p** | **N** | **q** |
| --- | --- | --- | --- | --- | --- |
| Blautia sp. SC05B48 | ↓ Higher values | -0.30 (0.10) | .001** | 79 | .127 |
| Faecalibacterium sp. I4-1-79 | ↓ Higher values | -0.44 (0.15) | .002** | 79 | .127 |
| Faecalibacterium sp. I2-3-92 | ↓ Higher values | -0.35 (0.12) | .002** | 79 | .127 |
| Bifidobacterium longum | ↓ Higher values | -0.53 (0.18) | .003** | 79 | .127 |
| Faecalibacterium sp. i25-0019-C1 | ↓ Higher values | -0.33 (0.12) | .003** | 79 | .127 |
| Blautia obeum | ↓ Higher values | -0.23 (0.09) | .003** | 79 | .127 |
| Coprococcus catus | ↓ Higher values | -0.26 (0.10) | .004** | 79 | .127 |
| Faecalibacterium sp. IP-3-29 | ↓ Higher values | -0.33 (0.13) | .005** | 79 | .127 |
| Faecalibacterium duncaniae | ↓ Higher values | -0.30 (0.12) | .005** | 79 | .127 |
| Anaerostipes hadrus | ↓ Higher values | -0.36 (0.14) | .005** | 79 | .127 |
| Faecalibacterium sp. IP-1-18 | ↓ Higher values | -0.31 (0.12) | .006** | 79 | .127 |
| Faecalibacterium prausnitzii | ↓ Higher values | -0.28 (0.12) | .007** | 79 | .143 |
| Faecalibacterium sp. I4-3-84 | ↓ Higher values | -0.26 (0.11) | .008** | 79 | .143 |
| Collinsella aerofaciens | ↓ Higher values | -0.46 (0.19) | .008** | 79 | .143 |
| Escherichia coli | ↑ Higher values | 0.83 (0.29) | .009** | 67 | .154 |
| Blautia hansenii | ↑ Higher values | 0.43 (0.14) | .011* | 78 | .168 |
| Bifidobacterium catenulatum | ↓ Higher values | -0.41 (0.18) | .013* | 77 | .189 |
| Faecalibacterium sp. i21-0019-B1 | ↓ Higher values | -0.27 (0.13) | .018* | 79 | .246 |
| Collinsella sp. i05-0019-G5 | ↓ Higher values | -0.18 (0.09) | .019* | 70 | .249 |
| Faecalibacterium sp. I3-3-89 | ↓ Higher values | -0.34 (0.16) | .020* | 79 | .249 |
| Intestinibacter bartlettii | ↓ Higher values | -0.51 (0.24) | .026* | 73 | .304 |
| Streptococcus thermophilus | ↓ Higher values | -0.52 (0.25) | .029* | 77 | .324 |
| Agathobaculum massiliense | ↑ Higher values | 0.29 (0.11) | .037* | 77 | .397 |
| Toutatisvirus toutatis | ↓ Higher values | -0.30 (0.16) | .038* | 60 | .397 |
| Anaerobutyricum hallii | ↓ Higher values | -0.17 (0.10) | .041* | 79 | .405 |
| Ruminococcus sp. SR1/5 | ↓ Higher values | -0.21 (0.12) | .043* | 79 | .409 |
| Lachnospira eligens | ↓ Higher values | -0.31 (0.17) | .045* | 79 | .414 |
| Subdoligranulum variabile | ↓ Higher values | -0.17 (0.10) | .047* | 79 | .414 |
| Parabacteroides merdae | ↑ Higher values | 0.34 (0.14) | .049* | 79 | .424 |

Notes. Notes. Tables summarize nominally significant (p < .05) results from exploratory MaAsLin3 mixed-effects models fitted to centred log-ratio–transformed species-level relative abundance data, with a random intercept for participant ID. N reflects the number of samples with non-zero abundance for each taxon. Direction arrows indicate higher (↑) or lower (↓) relative abundance with higher values of the predictor. False discovery rate–adjusted q-values are reported for transparency. p < .05*, p < .01**, p < .001***.

Table S14. Nominally significant taxa associated with Global sleep score

| **Taxon** | **Direction (Higher vs Lower Global sleep score)** | **β (SE)** | **p** | **N** | **q** |
| --- | --- | --- | --- | --- | --- |
| Megamonas funiformis | ↓ Higher values | -1.96 (0.49) | .007** | 14 | 1.000 |
| Lawsonibacter asaccharolyticus | ↑ Higher values | 0.31 (0.13) | .031* | 78 | 1.000 |
| Enterocloster bolteae | ↑ Higher values | 0.23 (0.09) | .031* | 78 | 1.000 |
| Flavonifractor plautii | ↑ Higher values | 0.25 (0.10) | .035* | 78 | 1.000 |
| Oscillibacter sp. PEA192 | ↑ Higher values | 0.19 (0.07) | .035* | 78 | 1.000 |
| Phascolarctobacterium sp. Marseille-Q4147 | ↓ Higher values | -0.10 (0.05) | .038* | 71 | 1.000 |
| Blautia hansenii | ↑ Higher values | 0.28 (0.12) | .041* | 77 | 1.000 |
| Dialister massiliensis | ↑ Higher values | 0.57 (0.21) | .043* | 14 | 1.000 |
| Phocaeicola vulgatus | ↑ Higher values | 0.19 (0.08) | .047* | 78 | 1.000 |

Notes. Tables summarize nominally significant (p < .05) results from exploratory MaAsLin3 mixed-effects models fitted to centred log-ratio–transformed species-level relative abundance data, with a random intercept for participant ID. N reflects the number of samples with non-zero abundance for each taxon. Direction arrows indicate higher (↑) or lower (↓) relative abundance with higher values of the predictor. False discovery rate–adjusted q-values are reported for transparency. p < .05*, p < .01**, p < .001***.

Table S15. Nominally significant taxa associated with MVPA (Moderate to Vigorous Physical Activity [mins])

| **Taxon** | **Direction (Higher vs Lower MVPA)** | **β (SE)** | **p** | **N** | **q** |
| --- | --- | --- | --- | --- | --- |
| Ruminococcus bicirculans (ex Wegman et al. 2014) | ↓ Higher values | -0.86 (0.26) | .003** | 74 | .697 |
| Adlercreutzia hattorii | ↓ Higher values | -0.42 (0.14) | .006** | 64 | .697 |
| Toutatisvirus toutatis | ↑ Higher values | 0.30 (0.11) | .009** | 57 | .697 |
| Adlercreutzia equolifaciens | ↓ Higher values | -0.30 (0.10) | .011* | 66 | .697 |
| Bacteroides sp. PHL 2737 | ↑ Higher values | 0.67 (0.29) | .030* | 18 | 1.000 |
| Bacteroides sp. DH3716P | ↑ Higher values | 0.23 (0.12) | .044* | 75 | 1.000 |
| Enterocloster asparagiformis | ↓ Higher values | -0.26 (0.12) | .050* | 75 | 1.000 |

Notes. Tables summarise nominally significant (p < .05) results from exploratory MaAsLin3 mixed-effects models fitted to centred log-ratio–transformed species-level relative abundance data, with a random intercept for participant ID. N reflects the number of samples with non-zero abundance for each taxon. Direction arrows indicate higher (↑) or lower (↓) relative abundance with higher values of the predictor. False discovery rate–adjusted q-values are reported for transparency. p < .05*, p < .01**, p < .001***.

**Table S16. Nominally significant taxa associated with Sedentary behaviour**

| **Taxon** | **Direction (Higher vs Lower Sedentary behaviour)** | **β (SE)** | **p** | **N** | **q** |
| --- | --- | --- | --- | --- | --- |
| Faecalibacillus intestinalis | ↑ Higher values | 0.32 (0.14) | .021* | 78 | 1.000 |

Notes. Tables summarise nominally significant (p < .05) results from exploratory MaAsLin3 mixed-effects models fitted to centred log-ratio–transformed species-level relative abundance data, with a random intercept for participant ID. N reflects the number of samples with non-zero abundance for each taxon. Direction arrows indicate higher (↑) or lower (↓) relative abundance with higher values of the predictor. False discovery rate–adjusted q-values are reported for transparency. p < .05*, p < .01**, p < .001***.

**Table S17**

***Sensitivity of Primary Stress-Condition Species-Level Associations Across Alternative Model Specifications***

**Panel A. Complete-pair, demographic, and lifestyle-adjusted models**

| **Taxon** | **Primary β** | **Complete-pair β (SE)** | **Age + sex + BMI β (SE)** | **Sleep β (SE)** | **Sedentary β (SE)** | **Vegetable β (SE)** | **Combined lifestyle β (SE)** |
| --- | --- | --- | --- | --- | --- | --- | --- |
| *Dialister massiliensis* | 0.52** | 0.52 (0.10)** | 0.52 (0.10)** | 0.53 (0.20) | 0.55 (0.14)* | 0.53 (0.12)* | 0.55 (0.27) |
| *Lawsonibacter asaccharolyticus* | 0.59** | 0.60 (0.18)** | 0.60 (0.18)** | 0.49 (0.20)* | 0.63 (0.19)** | 0.55 (0.19)** | 0.48 (0.21)* |
| *Duodenibacillus massiliensis* | -0.61** | -0.61 (0.21)** | -0.61 (0.21)** | -0.57 (0.28)* | -0.57 (0.23)* | -0.54 (0.22)* | -0.40 (0.37) |
| *Dialister hominis* | 0.48** | 0.48 (0.11)* | 0.48 (0.11)* | 0.46 (0.16)* | 0.55 (0.12)** | 0.49 (0.12)* | 0.57 (0.20)* |
| *Blautia obeum* | -0.23* | -0.22 (0.12)* | -0.22 (0.12)* | -0.18 (0.13) | -0.21 (0.12) | -0.25 (0.12)* | -0.20 (0.14) |
| *Oscillibacter hominis* | 0.27* | 0.27 (0.10)* | 0.27 (0.10)* | 0.21 (0.11) | 0.29 (0.10)* | 0.23 (0.10) | 0.19 (0.12) |
| *Oscillibacter sp. PEA192* | 0.28* | 0.28 (0.10)* | 0.28 (0.10)* | 0.19 (0.12) | 0.30 (0.11)* | 0.25 (0.11)* | 0.19 (0.12) |
| *Phascolarctobacterium sp. Marseille-Q4147* | -0.12* | -0.12 (0.07)* | -0.12 (0.07)* | -0.08 (0.08) | -0.09 (0.07) | -0.13 (0.07)* | -0.04 (0.09) |
| *Phocaeicola coprophilus* | -0.22* | -0.22 (0.11)* | -0.22 (0.11)* | -0.24 (0.13) | -0.21 (0.11)* | -0.19 (0.12) | -0.21 (0.14) |
| *Sutterella wadsworthensis* | -0.34* | -0.34 (0.15)* | -0.34 (0.15)* | -0.30 (0.18) | -0.37 (0.14)* | -0.34 (0.16)* | -0.32 (0.18) |

**Panel B. Sex-stratified and hormonal-contraceptive sensitivity models**

| **Taxon** | **Primary β** | **Female β (SE)** | **Male β (SE)** | **Female + hormonal contraception β (SE)** |
| --- | --- | --- | --- | --- |
| *Dialister massiliensis* | 0.52** | 0.36 (0.21) | 0.64 (0.08)** | — |
| *Lawsonibacter asaccharolyticus* | 0.59** | 0.35 (0.15)* | 0.97 (0.39)* | 0.35 (0.15)* |
| *Duodenibacillus massiliensis* | -0.61** | — | -0.80 (0.30)* | — |
| *Dialister hominis* | 0.48** | 0.37 (0.21) | 0.58 (0.09)* | — |
| *Blautia obeum* | -0.23* | -0.29 (0.10)** | -0.13 (0.25) | -0.29 (0.10)** |
| *Oscillibacter hominis* | 0.27* | 0.09 (0.10) | 0.53 (0.17)** | 0.09 (0.10) |
| *Oscillibacter sp. PEA192* | 0.28* | 0.03 (0.08) | 0.63 (0.20)** | 0.03 (0.08) |
| *Phascolarctobacterium sp. Marseille-Q4147* | -0.12* | -0.08 (0.07) | -0.21 (0.16) | -0.08 (0.07) |
| *Phocaeicola coprophilus* | -0.22* | -0.16 (0.13) | -0.29 (0.20) | -0.16 (0.13) |
| *Sutterella wadsworthensis* | -0.34* | 0.03 (0.10) | -0.68 (0.24)* | 0.03 (0.10) |

*Note.* Values are MaAsLin3 coefficients (β), with standard errors in parentheses for sensitivity models. Primary coefficients are shown for reference. The complete-pair model restricted analyses to participants with paired observations. The demographic model adjusted for age, sex, and BMI. Lifestyle models adjusted separately for global sleep quality, sedentary behaviour, and vegetable intake, or included all three variables simultaneously. Sex-stratified models were fitted separately in females and males; the hormonal-contraceptive model additionally adjusted for hormonal contraception within females. Asterisks indicate nominal significance (* p < .05, ** p < .01, *** p < .001) and are provided descriptively; sensitivity analyses were interpreted primarily according to stability in coefficient direction and approximate magnitude rather than retention of nominal significance. “—" indicates that an estimate was not available in the supplied robustness output. Complete model outputs, including FDR-adjusted q values, are provided in the online repository.
